# Osthole regulates N6‐methyladenosine‐modified TGM2 to inhibit the progression of rheumatoid arthritis and associated interstitial lung disease

**DOI:** 10.1002/mco2.219

**Published:** 2023-02-22

**Authors:** Xian Lin, Jian Chen, Cheng Tao, Lianxiang Luo, Juan He, Qingwen Wang

**Affiliations:** ^1^ Department of Rheumatism and Immunology Peking University Shenzhen Hospital Shenzhen China; ^2^ Institute of Immunology and Inflammatory Diseases Shenzhen Peking University‐The Hong Kong University of Science and Technology Medical Center; Shenzhen Key Laboratory of Inflammatory and Immunology Diseases Shenzhen China; ^3^ School of Pharmacy Guangdong Medical University Dongguan China; ^4^ The Marine Biomedical Research Institute Guangdong Medical University Zhanjiang China; ^5^ The Marine Biomedical Research Institute of Guangdong Zhanjiang Zhanjiang China

**Keywords:** interstitial lung disease, osthole, rheumatoid arthritis, TGM2

## Abstract

Rheumatoid arthritis (RA) is an inflammatory autoimmune disease, and RA interstitial lung disease (ILD) is a severe complication of RA. This investigation aims to determine the effect and underlying mechanism of osthole (OS), which could be extracted from *Cnidium*, *Angelica*, and *Citrus* plants and evaluate the role of transglutaminase 2 (TGM2) in RA and RA‐ILD. In this work, OS downregulated TGM2 to exert its additive effect with methotrexate and suppress the proliferation, migration, and invasion of RA‐fibroblast‐like synoviocytes (FLS) by attenuating NF‐κB signaling, resulting in the suppression of RA progression. Interestingly, WTAP‐mediated N6‐methyladenosine modification of TGM2 and Myc‐mediated WTAP transcription cooperatively contributed to the formation of a TGM2/Myc/WTAP‐positive feedback loop through upregulating NF‐κB signaling. Moreover, OS could downregulate the activation of the TGM2/Myc/WTAP‐positive feedback circuit. Furthermore, OS restrained the proliferation and polarization of M2 macrophages to inhibit the aggregation of lung interstitial CD11b^+^ macrophages, and the effectiveness and non‐toxicity of OS in suppressing RA and RA‐ILD progression were verified in vivo. Finally, bioinformatics analyses validated the importance and the clinical significance of the OS‐regulated molecular network. Taken together, our work emphasized OS as an effective drug candidate and TGM2 as a promising target for RA and RA‐ILD treatment.

## Graphical abstract

Osthole (OS) suppressed the positive feedback circuit transglutaminase 2 (TGM2)/Myc/WTAP‐facilitated NF‐κB signaling to attenuate the pathological phenotype of RA‐fibroblast‐like synoviocytes as well as impair the proliferation and polarization of CD11b^+^ M2 macrophages in the control of RA and RA‐interstitial lung disease (ILD) progression. OS might serve as an effective anti‐RA and anti‐RA ILD drug without toxicity, and TGM2 might be applied as a target for RA treatments.

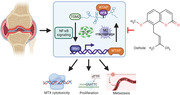

## 1 INTRODUCTION

Rheumatoid arthritis (RA), a kind of inflammatory autoimmune disease, is characterized by joint destruction and chronic synovitis and is accompanied by several complications.^1^ Therein, RA interstitial lung disease (ILD) was a severe complication and occurred in RA patients determined by high‐resolution computed tomography.^2^ Though the outcomes of RA patients with considerable morbidity and increased mortality could be somehow improved by disease‐modifying anti‐RA drugs, it is still urgent to resolve the high inefficient rate, high costs, and severe side effects of existing therapies.^3^ Methotrexate (MTX) is a well‐known disease‐modifying anti‐rheumatic drug, which is recommended as one of the first‐line therapeutic drugs for RA due to its potent efficacy.^2^ Though MTX benefits many RA patients, the mild to severe MTX‐associated multifarious side effects resulted in treatment discontinuation.^4^ Therefore, it is notable to investigate alternative drugs and targets for overcoming RA treatment failure and managing RA‐ILD. Fibroblast‐like synoviocytes (FLS) serve as the vital effector cells displaying a cancer‐like phenotype that evokes inflammatory and immune responses in RA.^5^ Since targeting RA‐FLS could inhibit RA progression,^6^, ^7^, ^8^ developing novel and effective drugs targeting FLS with less toxicity is a reasonable strategy for the management of RA.

In the past few decades, more and more natural compounds have exhibited their efficiency in treating RA.^9^ Our previous studies also demonstrated several natural compounds, such as sarsasapogenin,^5^ shikonin,^10^ and celastrol,^11^ that served as potential anti‐RA drugs. Osthole (OS) is an active ingredient that could be extracted from *Cnidium*, *Angelica*, and *Citrus* plants with low cost and presented anti‐inflammatory^12^, ^13^, ^14^ and immunosuppressive activities^15^, ^16^ in human diseases. Nevertheless, the therapeutical effect of OS on RA and RA‐ILD is not fully understood, and the potential role of OS in improving the sensitivity of traditional anti‐RA drugs is yet to be explored.

Transglutaminase 2 (TGM2) is a calcium‐dependent acyltransferase and affects many biological and pathological processes, such as chemotherapeutic sensitivity, apoptosis, inflammation, proliferation, epithelial‐mesenchymal transition, and stem cell renewal characteristics, and participates in the regulation of the tumor microenvironment.^17^ A previous report elucidated that TGM2 promoted the formation of invasive pseudopodia in FLS and was associated with cartilage destruction in RA.^18^ In addition, TGM2 was considered a marker of M2 macrophages^19^ and was shown to be an N6‐methyladenosine (m^6^A)‐modified gene that correlated with the abnormal synovial microenvironment in our previous study.^5^ However, the potential role of TGM2 in the progression of RA and RA‐ILD remains to be fully elucidated.

This exploration aims to investigate the therapeutic impact of OS and the effect of TGM2 on RA and RA‐ILD. Using primary RA‐FLS from RA patients and an immortalized RA‐FLS cell line, animal models, as well as available public databases, we measured the impact of OS on anti‐RA effect of MTX as well as proliferation and metastasis of RA‐FLS to determine its anti‐RA activity and detected the modulation of m^6^A‐modified TGM2 expression induced by OS. Particularly, we further uncovered the specific mechanisms of OS in controlling the progression of RA and RA‐ILD, laying a foundation for the clinical translation of OS as an effective drug and TGM2 as a promising target applied for RA and RA‐ILD treatments.

## 2 RESULTS

### 2.1 OS has an additive effect with MTX and suppresses proliferation, migration, and invasion in RA‐FLS

**FIGURE 1:**
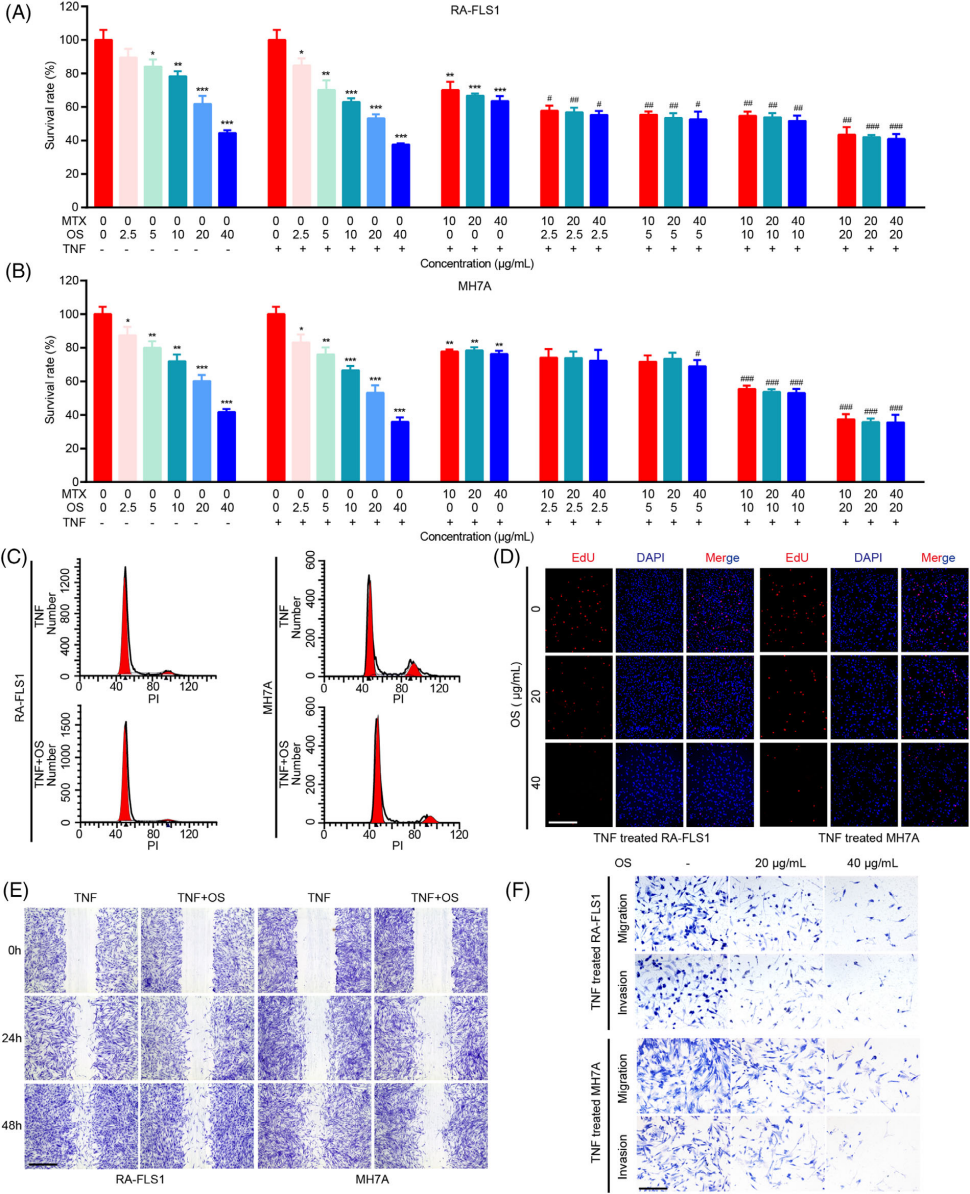
Osthole (OS) presents an additive effect with methotrexate (MTX) and inhibits proliferation, migration and invasion of rheumatoid arthritis‐fibroblast‐like synoviocytes (RA‐FLS). (A, B) Cells were incubated with indicated dosages of OS ( 0, 2.5, 5, 10, 20, and 40 μg/mL), MTX (0, 10, 20, and 40 μg/mL), or TNF (20 ng/mL) for 48 h, and cell viability of RA‐FLS1 (A) and MH7A (B) was measured by CCK8 assays. (C) The cell cycle analyses were conducted by flow cytometry in OS (0 and 20 μg/mL for 48 h)‐treated RA‐FLS and the controls. (D) EdU assays were applied for detecting DNA replication in OS (0, 20, and 40 μg/mL for 48 h)‐treated RA‐FLS and the controls. Scale bar: 50 μm. (E) Wound healing assays were adopted to present the migration ability of OS (0 and 20 μg/mL for 48 h)‐treated RA‐FLS and the controls. Scale bar: 125 μm. (F) Transwell assays were adopted to present the migration and invasion ability of OS (0, 20, and 40 μg/mL for 48 h)‐treated RA‐FLS and the controls. Scale bar: 50 μm. ^*^ *p* < 0.05, ^**^ *p* < 0.01, and ^***^ *P* < 0.001 versus TNF‐treated group. ^#^ *p* < 0.05, ^##^ *p* < 0.01, and ^###^ *p* < 0.001 versus TNF and MTX‐treated group.

To assess the anti‐RA effect of OS preliminarily, a cell viability assay was performed in RA‐FLS and showed that OS could inhibit the cell viability of primary RA‐FLS and an immortalized RA‐FLS cell line MH7A in a dosage‐dependent manner (Figure 1A,B). Interestingly, OS was capable of exhibiting an additive effect with MTX to exert their inhibitory effect on RA‐FLS growth in a dosage‐dependent manner as well (Figure 1A,B). Moreover, the inhibitory effect of OS on cell viability was magnified in RA‐FLS cultured in a median containing TNFα. Thus, further investigation was performed in RA‐FLS with a TNFα‐stimulated environment. Flow cytometry assays and EdU staining showed that OS suppressed DNA replication of RA‐FLS (Figures 1C,D and S1A,B). Confirming the suppressive role of OS in RA‐FLS proliferation through suppressing DNA replication, we then measured the impact of OS on RA‐FLS metastasis. The wound healing assay revealed that OS impaired the migration of RA‐FLS (Figures 1E and S1C). Furthermore, the transwell assay with or without Matrigel indicated that OS treatment weakened the migration and invasion capability of RA‐FLS (Figures 1F and S1D). These findings suggested that OS had an additive effect with MTX and alleviated cell proliferation, migration, and invasion in RA‐FLS.

### 2.2 RNA‐seq analyses reveal the potential regulation of TNF/NF‐κB signaling by OS in RA‐FLS

**FIGURE 2:**
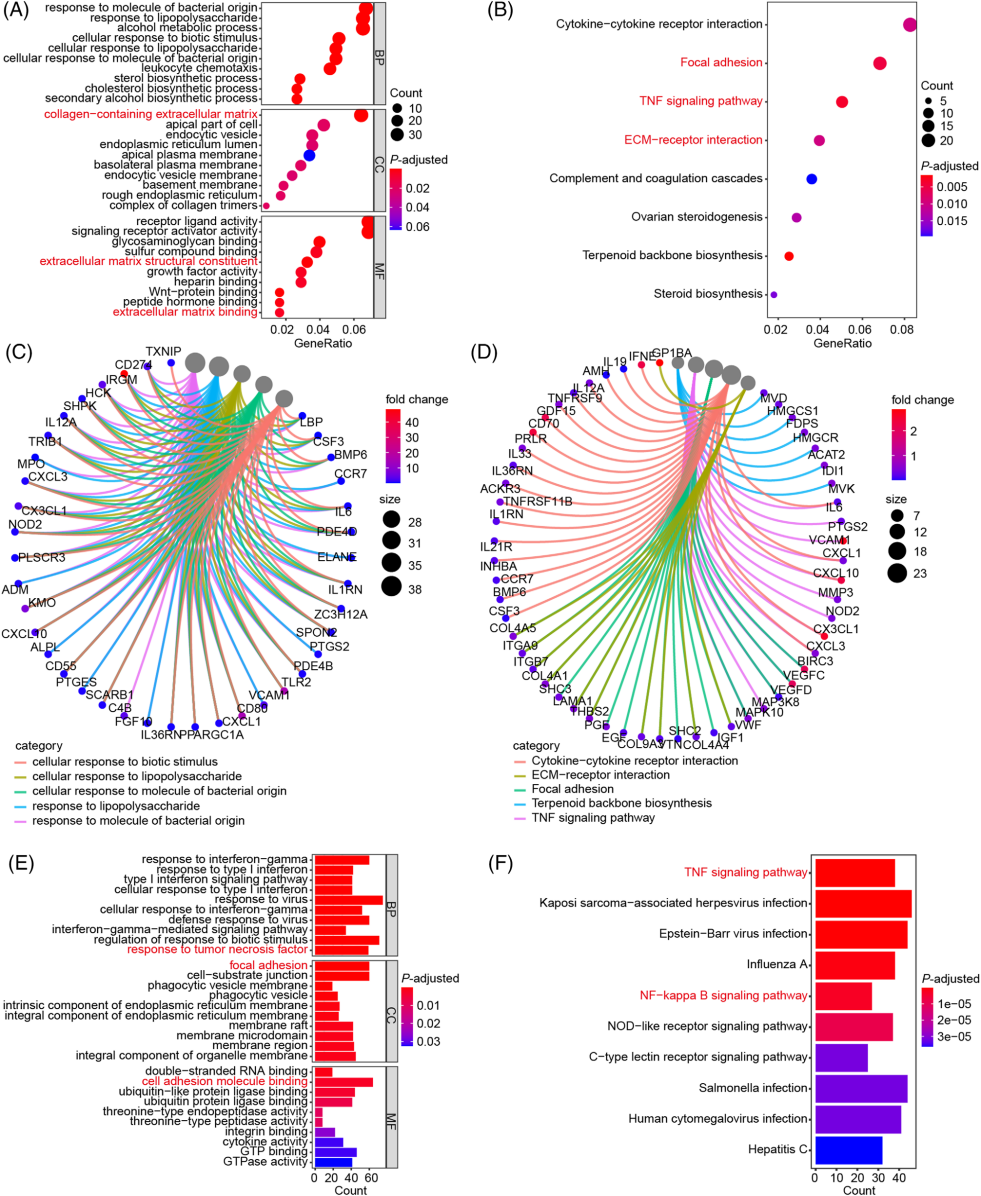
OS potentially regulates TNF/NF‐κB signaling based on RNA‐seq analyses. (A–D) Bubble‐plots and chords displaying the top enriched Gene Ontology (GO) (A, C) and Kyoto Encyclopedia of Genes and Genomes (KEGG) (B, D) terms according to our RNA‐seq data. RA‐FLS were cultured with 20 μg/mL of OS for 48 h. OS‐treated RA‐FLS and the controls were collected and used for RNA‐seq. (E, F) Bar‐plots showing the top enriched GO (E) and KEGG (F) terms according to RNA‐seq data of TNF‐induced RA‐FLS and the controls in GSE129486.

Subsequently, RNA‐seq transcriptome profiles of RA‐FLS were analyzed to achieve a better understanding of how OS mitigated the pathological phenotype of RA‐FLS. Therein, a total of 999 differentially expressed genes were obtained between OS‐treated RA‐FLS and the controls (fold change ≥ 2, *p* < 0.05). Gene set enrichment analysis (GSEA) with top Gene Ontology (GO) and Kyoto Encyclopedia of Genes and Genomes (KEGG) terms showed that OS was involved in modulating TNF signaling pathway and metastasis‐associated processes, including collagen‐containing extracellular matrix (ECM), extracellular matrix binding, matrix structural constituent, focal adhesion, ECM‐receptor interaction, and so on (Figure 2A–D). Since NF‐κB, PI3K‐AKT, and MAPK pathways could serve as downstream effectors of TNF signaling,^20^ we further explore the downstream signal of the TNF pathway in RA‐FLS. GSEA of differentially expressed genes in RA‐FLS after TNFα stimulation based on RNA‐seq data of GSE129486 dataset revealed the involvement of TNF signaling pathway, NF‐κB signaling pathway, response to TNF, focal adhesion, cell adhesion molecule (CAM) binding, and so on (Figures 2E,F and S2A,B). The findings indicated that OS might affect RA‐FLS functions by regulating TNF/NF‐κB signaling.

### 2.3 OS attenuates TGM2 expression to inhibit the pathological phenotype of RA‐FLS through modulating NF‐κB signaling

**FIGURE 3:**
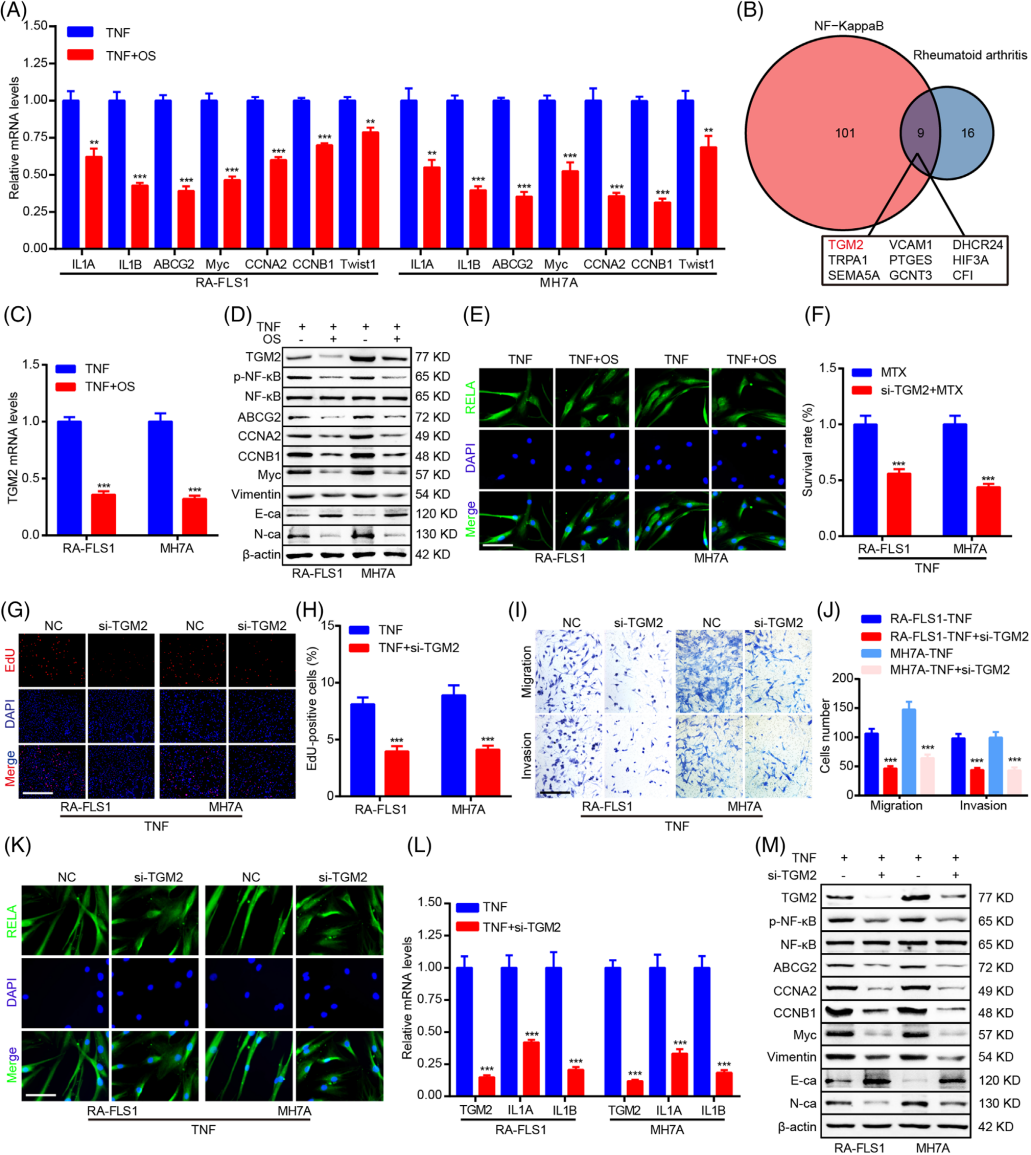
OS suppresses transglutaminase 2 (TGM2) expression to attenuate the pathological phenotype of RA‐FLS through modulating NF‐κB signaling. (A) qPCR detection of ILA, ILB, ABCG2, Myc, CCNA2, CCNB1, and Twist1 mRNA levels in RA‐FLS. (B) A Venn diagram analysis identifying genes associated with NF‐κB and RA signaling based on GSE109449 dataset. (C) qPCR detection of TGM2 mRNA levels in RA‐FLS. (D) Western blot measurement of TGM2, p‐NF‐κB, NF‐κB, ABCG2, CCNA2, CCNB1, Myc, Vimentin, N‐ca, E‐ca, and β‐actin protein levels in RA‐FLS. (E) Immunofluorescence staining exhibiting the impact of OS on RELA subcellular localization. Scale bar: 12.5 μm. (F) CCK8 assays were adopted to show the impact of TGM2 knockdown on the cell viability of MTX‐treated RA‐FLS. (G, H) EdU assays were applied to detect the effect of TGM2 knockdown on DNA replication of RA‐FLS. Scale bar: 50 μm. (I, J) Transwell assays were adopted to determine the impact of TGM2 knockdown on RA‐FLS migration and invasion ability. Scale bar: 50 μm. (K) Immunofluorescence staining was performed to exhibit the impact of TGM2 knockdown on RELA subcellular localization. Scale bar: 12.5 μm. (L) qPCR detection of TGM2, IL1A, and IL1B mRNA levels in RA‐FLS. (M) Western blot measurement of TGM2, p‐NF‐κB, NF‐κB, ABCG2, CCNA2, CCNB1, Myc, vimentin, N‐ca, E‐ca, and β‐actin protein levels in RA‐FLS. ^**^ *p* < 0.01 and ^***^ *p* < 0.001 versus TNF‐treated group.

The role of NF‐κB signaling in the anti‐RA effect of OS was further investigated to confirm the results of bioinformatics analyses. The qPCR assay indicated that OS inhibited the expression of known targets and downstream effectors of NF‐κB signaling, including IL1A, IL1B, ABCG2, Myc, CCNA2, CCNB1, and Twist1 in RA‐FLS (Figure 3A). Next, we further explored the potential downstream effector that was responsible for the inhibitory role of OS in NF‐κB signaling. First, the NF‐κB signaling and RA activity of a single sample were calculated by single sample GSEA (ssGSEA) based on RNA‐seq data of RA‐FLS in GSE109449 dataset. Second, correlation analyses were conducted to elucidate specific genes correlated with the NF‐κB signaling and RA activity (|*r*| ≥ 2.0, *p* < 0.05). Finally, nine genes were shown to be associated with NF‐κB signaling and RA (Figure 3B). Therein, TGM2 ranked first among the identified nine genes and was a differentially expressed gene among the top 50 list in RA‐FLS after OS treatment based on our RNA‐seq data (Figure S3A,B). Moreover, qPCR verified that OS impaired TGM2 expression (Figure 3C), and western blot confirmed that OS suppressed the protein expression of TGM2, p‐NF‐κB, ABCG2, CCNA2, CCNB1, Myc, Vimentin, and N‐ca and promoted E‐ca protein expression (Figure 3D). TGM2 was shown to interact with NF‐κB components to trigger nuclear translocation of a subunit of NF‐κB, namely, RELA.^7^ We then performed immunofluorescence to test the effect of OS on TGM2‐mediated RELA intracellular localization and found that OS restrained the nuclear translocation of RELA, further indicating the inhibitory effect of OS on NF‐κB signaling pathway (Figure 3E).

To further discuss the role of TGM2 in RA‐FLS, TGM2 siRNAs were adopted to silence TGM2 expression. The cell viability, EdU staining, and transwell assays displayed that TGM2 knockdown further inhibited the cell viability of MTX‐treated RA‐FLS and impaired proliferation, migration, and invasion of RA‐FLS (Figure 3F–J). Moreover, TGM2 depletion downregulated the nuclear translocation of RELA detected by immunofluorescence (Figure 3K). Furthermore, qPCR and western blot assays confirmed the silence of TGM2 and further indicated the downregulation of NF‐κB signaling (p‐NF‐κB, IL1A, and IL1B); the inhibition of the expression of genes associated with MTX sensitivity (ABCG2), proliferation (CCNA2, CCNB1, and Myc) and metastasis (Vimentin and N‐ca); and the upregulation of metastasis‐associated gene (E‐ca) expression induced by TGM2 knockdown (Figure 3L,M). Moreover, TGM2 overexpression induced a stimulatory effect on the pathological phenotype of RA‐FLS (Figure S4A–E).

**FIGURE 4:**
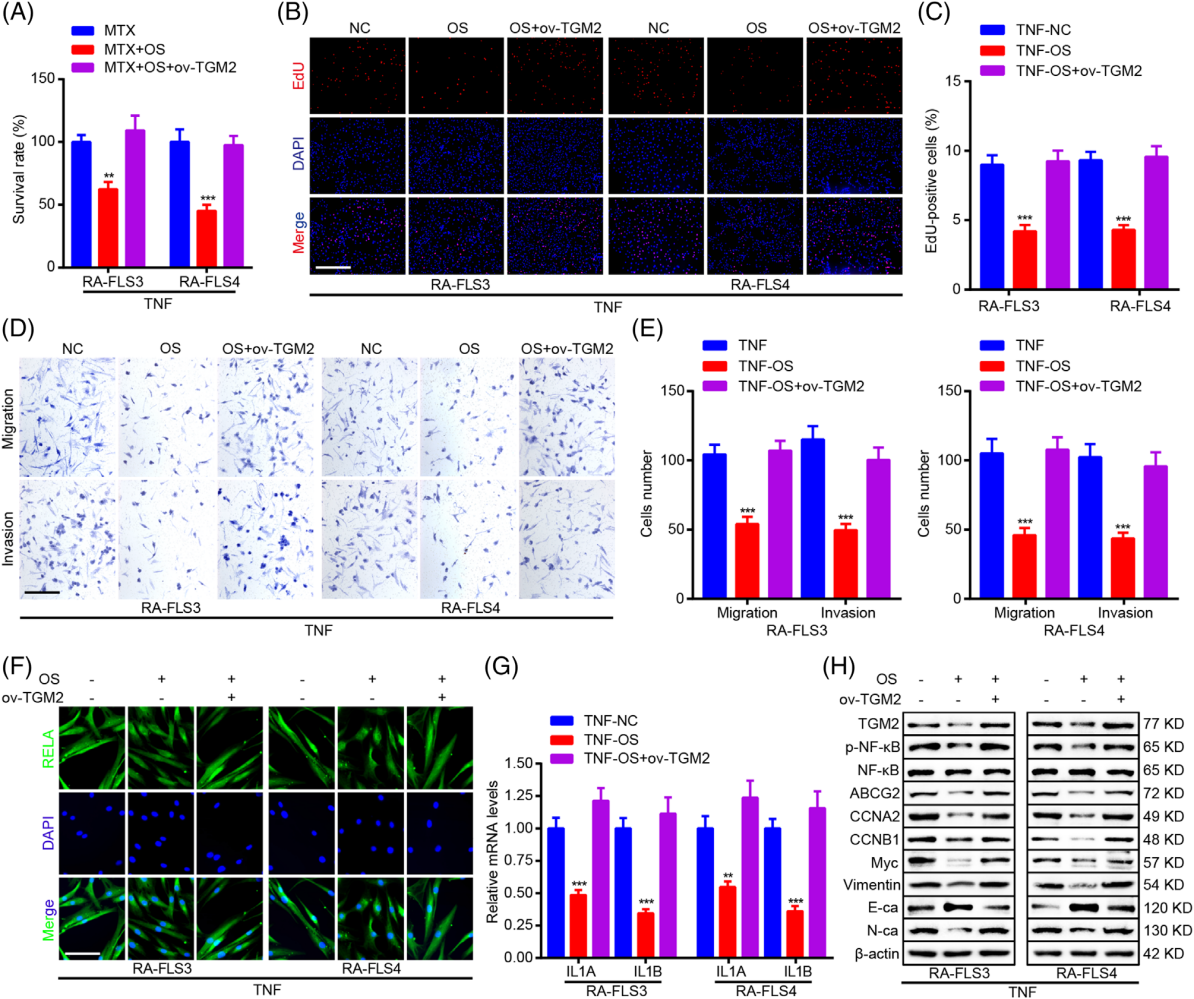
TGM2 mediates the inhibitory role of OS in the pathological phenotype of RA‐FLS. (A) CCK8 assays were adopted to show the impact of OS and TGM2 overexpression on the cell viability of MTX‐treated RA‐FLS. (B, C) EdU assays were applied for measuring the impact of OS and TGM2 overexpression on DNA replication of RA‐FLS. Scale bar: 50 μm. (D, E) Transwell assays were adopted to reveal the impact of OS and TGM2 overexpression on RA‐FLS migration and invasion ability. Scale bar: 50 μm. (F) Immunofluorescence staining was performed to exhibit the impact of OS and TGM2 overexpression on RELA subcellular localization. Scale bar: 12.5 μm. (G) qPCR detection of IL1A and IL1B mRNA levels in RA‐FLS. (H) Western blot measurement of TGM2, p‐NF‐κB, NF‐κB, ABCG2, CCNA2, CCNB1, Myc, vimentin, N‐ca, E‐ca, and β‐actin protein levels in RA‐FLS. ^**^ *p* < 0.01 and ^***^ *p* < 0.001 versus TNF‐treated group

Subsequently, the mediation role of TGM2 in the anti‐RA effect of OS was revealed in RA‐FLS. In another two primary RA‐FLS, the cell viability, EdU staining, and transwell assays validated that OS presented an additive effect with MTX and suppressed proliferation, migration, and invasion of RA‐FLS (Figure 4A–E). Moreover, immunofluorescence, qPCR, and western blot assays confirmed that OS impaired TGM2 expression, NF‐κB signaling, and NF‐κB downstream effectors expression and modulated MTX sensitivity, proliferation, and metastasis‐associated genes expression (Figure 4F–H). In addition, the anti‐RA effect of OS in RA‐FLS could be reversed by TGM2 overexpression (Figure 4A–H).

Above all supported that OS inhibited the pathological phenotype of RA‐FLS through attenuating TGM2‐mediated NF‐κB signaling.

### 2.4 A TGM2/Myc/WTAP‐positive feedback loop contributed to the inhibitory impact of OS on NF‐κB signaling pathway

**FIGURE 5:**
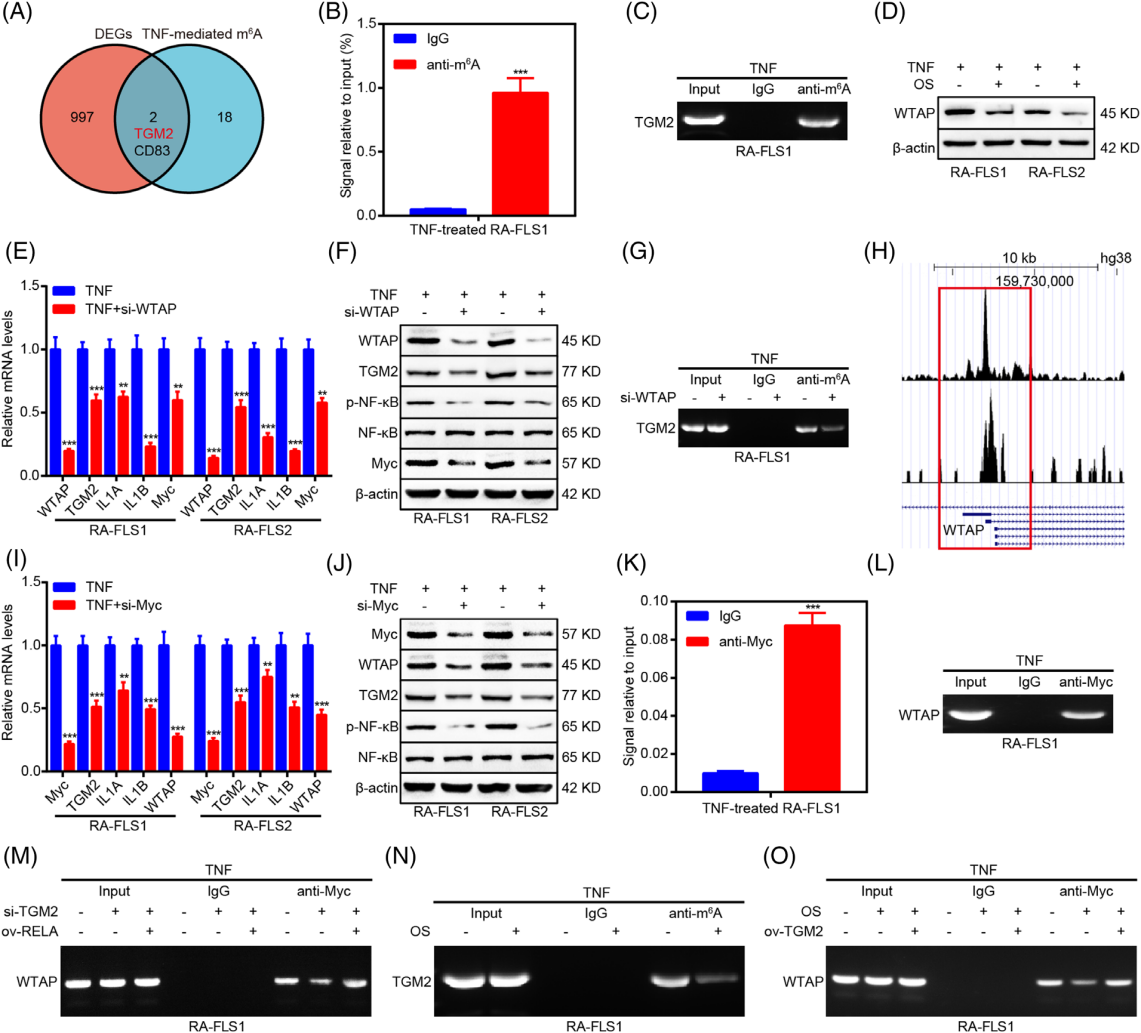
OS impairs the activity of a TGM2/Myc/WTAP‐positive feedback loop mediated by NF‐κB signaling. (A) A Venn diagram analysis revealing genes associated with TNF‐mediated N6‐methyladenosine (m6A) modification. (B, C) methylated RNA immunoprecipitation (MeRIP) assays combined with qPCR (B) and PCR (C) were conducted to detect m6A modification of TGM2 mRNA. (D) Western blot analyses were adopted to explore the impact of OS on WTAP protein levels in RA‐FLS. (E) qPCR assays were applied to reveal the impact of WTAP knockdown on TGM2, IL1A, IL1B, and Myc mRNA levels in RA‐FLS. (F) Western blot analyses were adopted to detect the impact of WTAP depletion on TGM2, p‐NF‐κB, NF‐κB, and Myc protein levels in RA‐FLS. (G) MeRIP assays combined with PCR displaying the effect of WTAP knockdown on m^6^A modification of TGM2 mRNA. (H) Bioinformatics analyses elucidating a Myc binding site within the promoter region of WTAP based on Cistrome Data Browser. (I) qPCR assays were applied to reveal the impact of Myc knockdown on TGM2, IL1A, IL1B, and WTAP mRNA levels in RA‐FLS. (J) Western blot analyses were adopted to detect the impact of Myc depletion on WTAP, TGM2, p‐NF‐κB, and NF‐κB protein levels in RA‐FLS. (K, L) Chromatin immunoprecipitation (ChIP) assays combined with qPCR (K) and PCR (L) were applied to measure Myc‐mediated WTAP transcription. (M) ChIP assays combined with PCR showing the impact of TGM2 and RELA on Myc‐mediated WTAP transcription. (N) MeRIP assays combined with PCR displaying the impact of OS on *TGM2* mRNA m^6^A modification. (O) ChIP combined with PCR assays showing the impact of OS and TGM2 on Myc‐mediated WTAP transcription. ^**^ *p* < 0.01 and ^***^ *p* < 0.001 versus TNF‐treated group.

Our previous work demonstrated the important role of TGM2 mRNA m^6^A modification in RA.^5^ Thus, we focused on whether TGM2 mRNA m^6^A modification was responsible for the role of OS in RA‐FLS and compared differentially expressed genes in RA‐FLS after OS treatment based on our RNA‐seq data with the top 20 genes that were associated with TNFα‐mediated m^6^A modification in RA‐FLS based on a previous study.^21^ Interestingly, TGM2 was identified as a gene affected by m^6^A modification according to the intersection (Figure 5A), and methylated RNA Immunoprecipitation (MeRIP) verified TGM2 as an m^6^A‐modified gene according to the m^6^A site identified in our previous work (Figure 5B,C).^5^ We then discussed the m^6^A regulator that was responsible for TGM2 mRNA m^6^A modification. Intriguingly, WTAP was suggested as a potential m^6^A regulator modulating TGM2 mRNA m^6^A modification in the m6a2Target database (http://m6a2target.canceromics.org/) and was shown as a TNFα‐inducible gene in RA‐FLS.^21^ In addition, OS could downregulate WTAP expression, and WTAP knockdown caused the reduction of TGM2 expression as well as the downregulation of NF‐κB signaling (p‐NF‐κB, IL1A, IL1B, and Myc; Figure 5D–F). MeRIP further revealed that WTAP knockdown resulted in the decrease of TGM2 mRNA m^6^A modification (Figure 5G). These findings suggested that WTAP‐mediated TGM2 mRNA m^6^A modification contributed to the regulation of TGM2 expression and NF‐κB signaling in RA‐FLS.

Subsequently, we determined how OS regulated WTAP expression. Interestingly, a Myc binding site was identified in the promoter region of WTAP as per Cistrome Data Browser (http://cistrome.org/db/#/ ; Figure 5H). In addition, Myc knockdown also led to the reduction of WTAP and TGM2 expression as well as the downregulation of NF‐κB signaling (p‐NF‐κB, IL1A, and IL1B; Figure 5I,J). Chromatin immunoprecipitation (ChIP) assays further elucidated that Myc bounded to the promoter region of WTAP (Figure 5K,L). More importantly, ChIP assays showed that TGM2 knockdown impaired Myc‐mediated WTAP transcription, and the inhibitory effect induced by TGM2 knockdown could be restored by RELA overexpression (Figure 5M), suggesting the formation of the TGM2/Myc/WTAP‐positive feedback loop via NF‐κB signaling. Furthermore, MeRIP and ChIP assays indicated that OS downregulated *TGM2* mRNA m^6^A modification and Myc‐mediated WTAP transcription (Figure 5N,O), and the suppressive role of OS in Myc‐mediated WTAP transcription could be reversed by TGM2 overexpression. When discussing the real target of OS, we predicted RELA as a target of OS by molecular docking (Figure S5A,B). Moreover, cellular thermal shift assay indicated that RELA might be a target of OS (Figure S5C).

All mentioned above collectively supported that OS might target NF‐κB signaling to modulate *TGM2* mRNA m^6^A modification and suppress the TGM2/Myc/WTAP‐positive feedback loop.

### 2.5 OS cooperates with MTX to suppress RA progression in vivo

**FIGURE 6:**
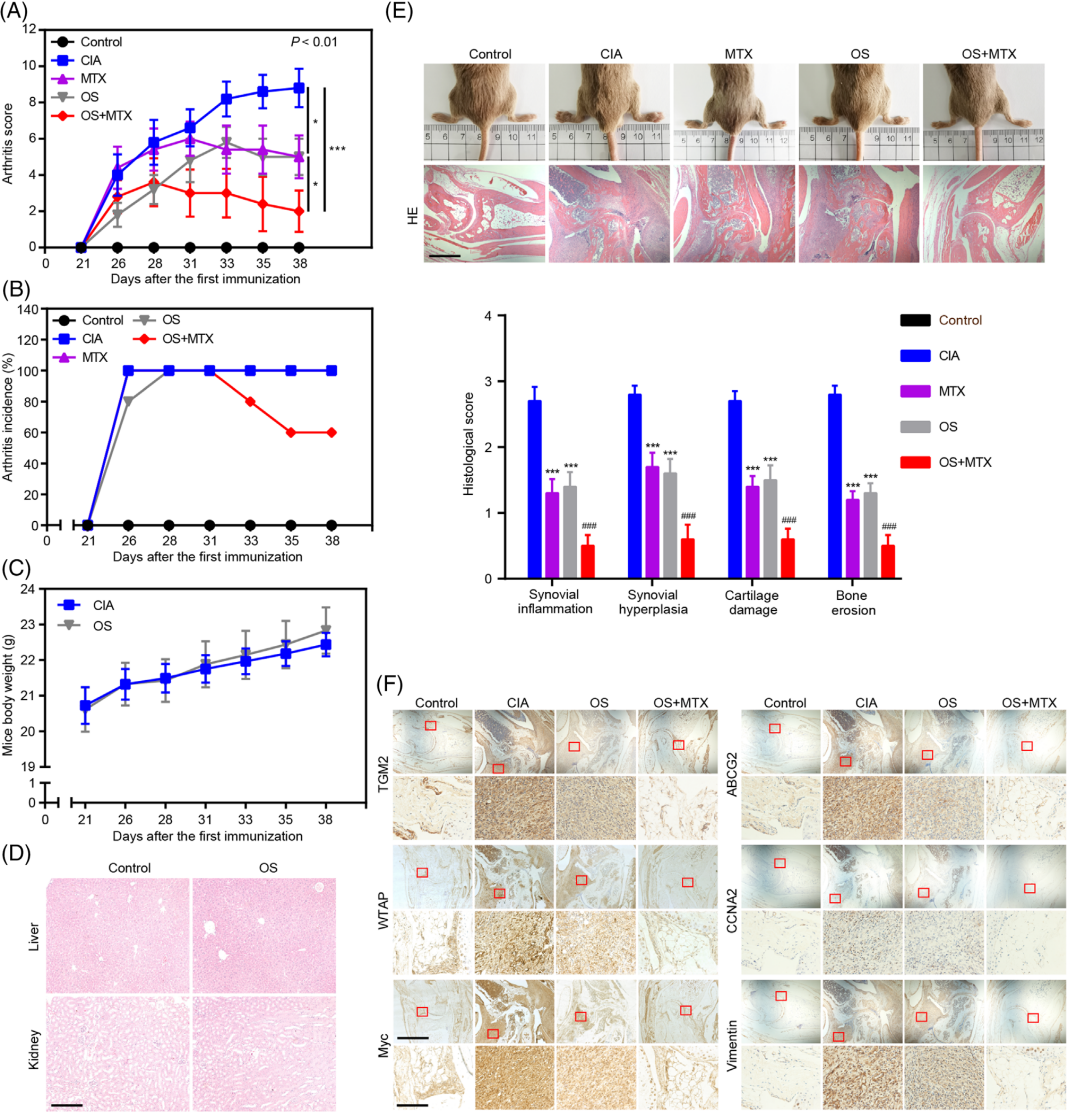
OS cooperates with MTX to inhibit RA progression in vivo. The collagen‐induced arthritis (CIA) mice model was constructed and randomly divided into control, CIA, MTX, OS, and OS combined with MTX treatment groups (*n* = 10 per group). (A, B) The impacts of OS and MTX on arthritis score (A) and incidence of arthritis (B) in CIA models. (C) The measurement of mice body weight in CIA and OS group. (D) Representative histopathological images of mice livers and kidneys in OS‐treated and the control groups. Scale bar: 50 μm. (E) Gross and H&E staining view of mice joints in CIA models exhibiting the impacts of OS and MTX on arthritis. Synovial inflammation, synovial hyperplasia, cartilage damage, and bone erosion were evaluated in the mice joints. Scale bar: 125 μm. (F) Representative images (upper scale bar: 125 μm and below scale bar: 12.5 μm) of immunohistochemistry staining exhibiting the impacts of OS and MTX on TGM2, WTAP, Myc, ABCG2, CCNA2, and vimentin expression in synovial tissue of joints. ^*^ *p* < 0.05, and ^***^ *p* < 0.001 versus CIA group. ^###^ *p* < 0.001 versus MTX‐treated group.

To verify the anti‐RA effect of OS in vivo, we constructed a collagen‐induced arthritis (CIA) mouse model. The arthritis scores of the model showed that three times per week treatments of OS (50 mg/kg) and the positive control drug MTX (23 mg/kg) efficiently relieved the swelling of mice joints, and the mice joints swollen were further alleviated by the treatment of OS in combination with MTX, compared to those treated with OS or MTX alone (Figure 6A). Interestingly, the combination of OS and MTX treatments could somehow cure arthritis (Figure 6B). Moreover, anti‐type II collagen IgG autoantibodies and inflammation‐related cytokine TNFα decreased in the serum of OS‐treated CIA mice, compared to that in CIA mice, and the effect was enlarged in mice receiving the treatment of OS in combination with MTX (Figure S6A,B). As for in vivo toxicity detection, no difference in the mice body weight and the histopathological changes was observed in the liver and kidney, which serve as the main organ for drug metabolism of mice intraperitoneally injected with OS, suggesting that OS had no obvious toxicity in vivo (Figure 6C,D). Moreover, histopathological changes, including synovial inflammation, synovial hyperplasia, cartilage damage, and bone erosion in the mice joints further suggested the anti‐RA effect of OS and the synergistic anti‐RA effect of OS and MTX in the CIA model (Figure 6E). In addition, immunohistochemistry staining showed the attenuated expression of TGM2, WTAP, Myc, ABCG2, CCNA2, and vimentin induced by OS in CIA models (Figure 6F), further verifying the regulatory role of OS in TGM2‐mediated pathological phenotype of RA‐FLS. The above data indicated that OS might be an effective drug candidate for RA treatments without obvious side effects.

### 2.6 OS inhibits RA‐associated ILD by downregulating the aggregation of M2 macrophage

**FIGURE 7:**
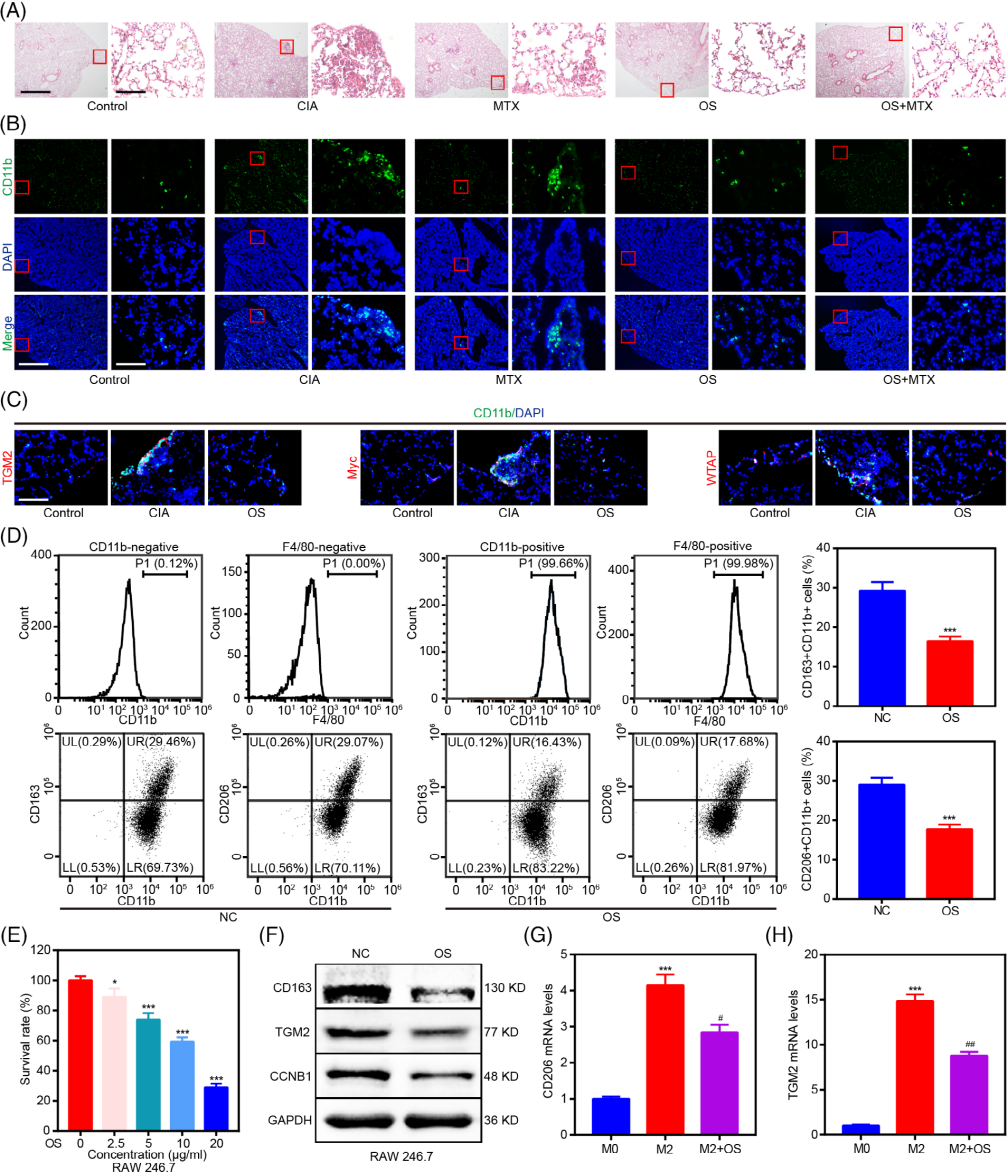
OS suppresses RA‐associated interstitial lung disease (ILD) by downregulating the aggregation of M2 macrophages. (A) Representative images (left scale bar: 125 μm and right scale bar: 12.5 μm) of H&E staining displaying the impact of OS and MTX on the generation of subpleural inflammation in CIA models. (B) Representative images (left scale bar: 125 μm and right scale bar: 12.5 μm) of immunofluorescence staining displaying the impact of OS and MTX on the aggregation of CD11b^+^ interstitial macrophage in the subpleural inflammation area of the CIA mouse model. (C) Representative images (scale bar: 12.5 μm) of immunofluorescence staining displaying the impact of OS on TGM2, Myc, and WTAP expression of CD11b^+^ interstitial macrophage in the subpleural inflammation area of the CIA mouse model. (D) Flow cytometry indicating the effect of OS on the percentage of CD163^+^CD11b^+^ and CD206^+^CD11b^+^ RAW 246.7 cells. (E) RAW 246.7 cells were exposed to OS (0, 2.5, 5, 10, and 20 μg/mL) for 48 h, and cell viability was measured by CCK8 assays. (F) Western blot analyses were applied for elucidating the impact of OS on CCNB1, CD163, and TGM2 protein levels in RAW 246.7 cells. (G, H) qPCR assays were applied to reveal the impact of OS on CD206 (G) and TGM2 (H) in IL4 and IL13‐induced M2 macrophage differentiated from THP‐1. ^*^ *p* < 0.05 and ^***^ *p* < 0.001 versus control group. ^#^ *p* < 0.05 and ^##^ *p* < 0.01 versus M2 group.

Since RA‐associated ILD is one of the major complications leading to death in RA patients,^22^ the effect of OS on RA‐ILD was then investigated in CIA models. Histopathological staining showed the generation of subpleural inflammation in CIA models, and OS could alleviate RA‐associated ILD in CIA models (Figure 7A). Moreover, immunofluorescence staining revealed the aggregation of CD11b^+^ interstitial macrophage in the subpleural inflammation area, and OS could mitigate the aggregation of CD11b^+^ interstitial macrophage in CIA models (Figure 7B). However, the aggregation of CD11b^+^ macrophages could still be detected in lungs of MTX‐treated group, indicating the inhibitory effect of OS in RA‐ILD was superior to that of MTX. In addition, immunofluorescence staining showed that OS could downregulate TGM2, Myc, and WTAP expression of CD11b^+^ interstitial macrophage in lungs of CIA models, supporting that the effects of OS via RA‐FLS contributed to its protective effects of RA‐ILD. (Figure 7C).

Next, we tested the role of OS in macrophages through in vitro studies. First, flow cytometry was conducted, and it indicated that RAW 246.7 cells were CD11b^+^F4/80^+^ macrophages. In addition, OS lowered the percentage of CD163^+^CD11b^+^ and CD206^+^CD11b^+^ RAW 246.7 cells (Figure 7D). Then, OS was shown to suppress the proliferation of CD11b^+^ RAW 246.7 cells in a dosage‐dependent manner (Figure 7E). Moreover, western blot verified that OS inhibited the expression of M2 macrophage markers (CD163 and TGM2) and the proliferation‐associated gene (CCNB1) in CD11b^+^ RAW 246.7 cells (Figure 7F). Finally, OS was revealed to impair the expression of M2 macrophage markers (CD206 and TGM2) in IL4 and IL13‐induced M2 macrophages differentiated from THP‐1 (Figure 7G,H).

These data collectively demonstrated that OS reduced the proliferation and polarization of CD11b^+^ M2 macrophages to improve RA‐associated ILD.

### 2.7 Bioinformatics analysis emphasizes the significance of molecular regulatory network regulated by OS

**FIGURE 8:**
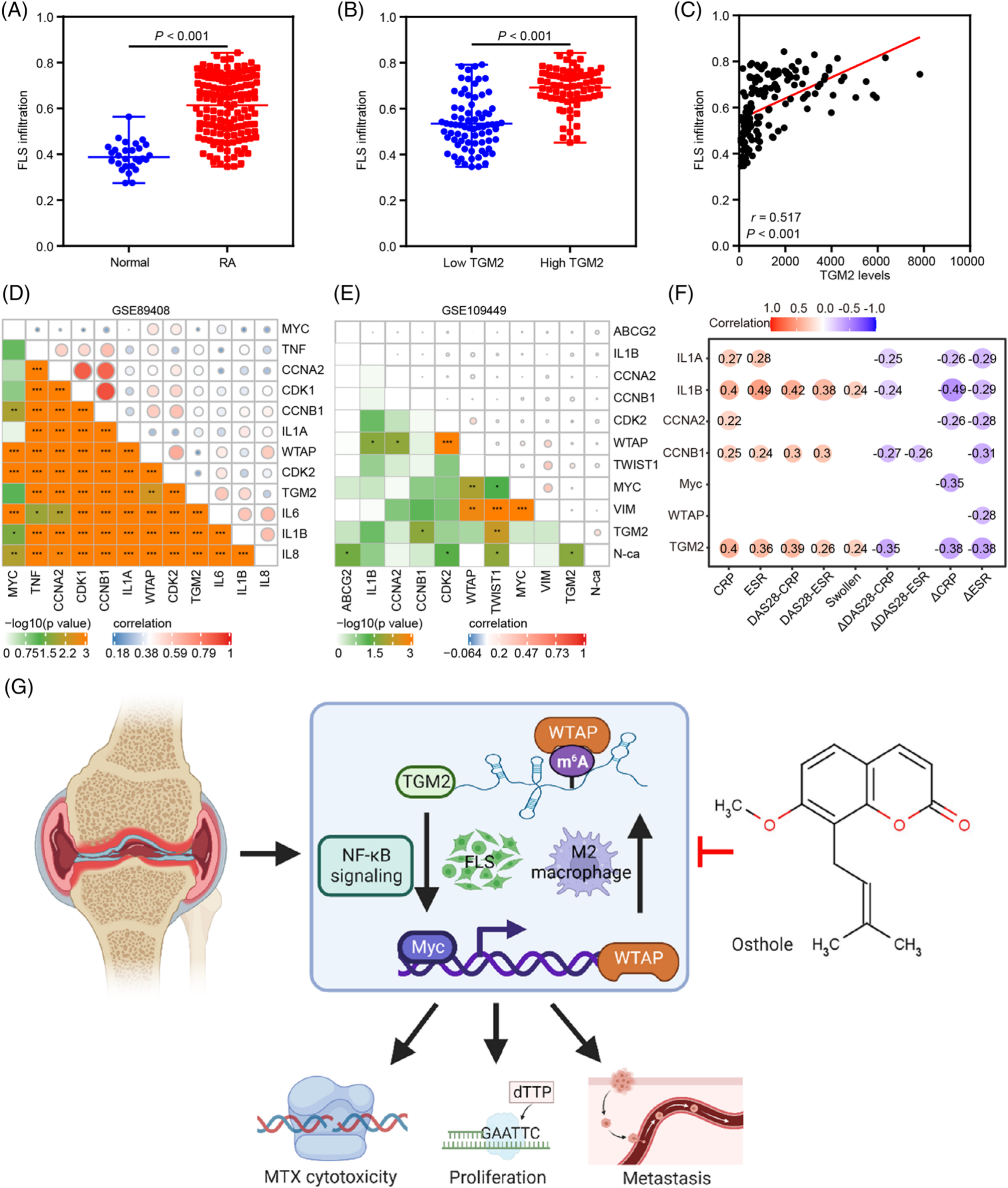
Bioinformatics analysis emphasizes the significance of the molecular regulatory network regulated by OS. (A) The differential distribution of FLS infiltration calculated by single sample gene set enrichment analysis (ssGSEA) between RA patients and normal controls based on GSE89408 dataset. (B) The differential distribution of FLS infiltration calculated by ssGSEA in RA patients with low and high TGM2 levels based on GSE89408 dataset. (C) The correlation analysis displaying the relationship between TGM2 levels and FLS infiltration calculated by ssGSEA in RA patients as per GSE89408 dataset. (D) The correlation analyses displaying the relationship among TGM2, Myc, WTAP, TNF, CCNA2, CCNB1, CDK1, CDK2, IL1A, IL1B, IL6, and IL8 levels as per GSE89408 dataset. (E) The correlation analyses displaying the relationship among TGM2, Myc, WTAP, ABCG2, CCNA2, CCNB1, CDK2, IL1B, Twist1, vimentin, and N‐ca levels as per GSE109449 dataset. (F) The correlation analyses presenting the relationship between the severity of RA disease (DAS28‐CRP, DAS28‐ESR, CRP, ESR, and joint swollen), therapy response (delta DAS28‐CRP, delta DAS28‐ESR, delta CRP, and delta ESR), and the levels of TGM2, Myc, WTAP, IL1A, IL1B, CCNA2, and CCNB1 based on PEAC RNA‐seq database. (G) Working model indicating the modulation of OS‐downregulated TGM2/Myc/WTAP‐positive feedback circuit in anti‐RA effect of MTX as well as proliferation and metastasis of RA‐FLS through modulating NF‐κB signaling. This graphical abstract was generated by applying the Biorender website (https://biorender.com/).

Bioinformatics analyses were subsequently performed to validate our findings. The data obtained from GSE89408 dataset were applied for calculating FLS infiltration by ssGSEA. Differential analyses showed that RA patients were characterized with more FLS infiltration than normal controls (Figure 8A), and RA patients in the high TGM2 group were infiltrated with more FLS (Figure 8B). Correlation analyses further indicated a positive association between TGM2 expression and FLS infiltration in RA patients (Figure 8C). The positive correlations among TGM2, Myc, WTAP, TNF, IL1A, IL1B, IL6, IL8, ABCG2, CCNA2, CCNB1, CDK1, CDK2, vimentin, Twist1, and N‐ca were further elucidated according to RNA‐seq data of synovial tissue and RA‐FLS in GSE89408 and GSE109449 datasets, respectively (Figure 8D,E). Importantly, correlation analyses indicated a positive association between the severity of RA disease (DAS28‐CRP, DAS28‐ESR, CRP, ESR, and joint swollen) and the expression of TGM2, Myc, WTAP, IL1A, IL1B, CCNA2, and CCNB1 as well as the negative correlation between therapy response (delta DAS28‐CRP, delta DAS28‐ESR, delta CRP, and delta ESR) and TGM2, Myc, WTAP, IL1A, IL1B, CCNA2, and CCNB1 expression (Figure 8F) based on the PEAC RNA‐seq database. GSEA as per GSE109449 dataset further indicated that RA‐FLS with high TGM2 expression was involved in the positive modulation of TNF/NF‐κB, inflammatory response, chemokine, DNA replication, cell cycle, and CAMs signaling pathways (Figure S7). These findings further emphasized the significance of the molecular regulatory network regulated by OS in RA progression.

## 3 DISCUSSION

In recent years, many compounds with promising anti‐RA activities isolated from natural products have drawn considerable attention.^23^, ^24^ In the current study, we took advantage of the promising applications of a coumarin‐derived active ingredient OS that exhibited anti‐RA effects by regulating proliferation, migration, and invasion of RA‐FLS via downregulating TGM2‐mediated signaling and presented anti‐RA‐ILD effects via attenuating the proliferation and polarization of CD11b^+^ M2 macrophage. Importantly, the anti‐RA and RA‐ILD activities of OS as well as the additive effect of OS in combination with MTX were validated in the CIA mouse model. Interestingly, the clinical significance of the OS‐regulated molecular network emphasized OS as an effective drug candidate and identified TGM2 as a promising target for RA treatment.

Previous studies reported the potential anti‐RA effect of OS.^25^, ^26^ In addition, OS has an inhibitory impact on cell proliferation, migration, and invasion.^27^, ^28^ In the present investigation, we found that OS not only inhibited RA‐FLS proliferation, migration, and invasion but also showed an additive effect with MTX. In the subsequent study, we showed that OS reduced NF‐κB signaling activity to exert its anti‐RA effect, which was consistent with another report indicating the role of NF‐κB signaling in regulating RA‐FLS proliferation, migration, and invasion.^29^ Furthermore, OS downregulated the expression of IL1A, IL1B, Myc, ABCG2, and vimentin, which are known NF‐κB targets,^30^, ^31^ as well as CCNA2, CCNB1, Twist1, and N‐ca, which are known downstream effectors of NF‐κB,^32^, ^33^, ^34^ and caused the elevation of E‐ca expression. Moreover, ABCG2 contributed to MTX resistance in RA.^35^, ^36^ The alleviation of the pathological phenotype of RA‐FLS and the enhanced anti‐RA effect of MTX induced by OS suggested that OS could effectively delay RA progression.

TGM2 was shown to enhance RA‐FLS invadopodia formation and cartilage breakdown in CIA models.^18^ In this investigation, TGM2 was identified as a downstream effector of OS. Moreover, we further demonstrated that TGM2 augmented NF‐κB signaling and the downstream effectors of NF‐κB, thus impairing the anti‐RA effect of MTX as well as augmenting proliferation, migration, and invasion of RA‐FLS. The findings that TGM2 played a stimulatory role in NF‐κB signaling were consistent with a previous report.^37^ Previous studies well established that TGM2 catalyzed the formation of covalent bonds to form the polymerization of IκBα and subsequently inhibited the interplay between IκBα and RELA as well as facilitated the nuclear translocation of RELA, thus activating NF‐κB signaling.^22^, ^38^ In our investigation, we also confirmed that TGM2 stimulated RELA nuclear translocation, further inducing the activation of NF‐κB signaling in RA‐FLS. Furthermore, we verified that TGM2 mediated the anti‐RA activity of OS through regulating NF‐κB signaling, suggesting TGM2 was a promising target for RA treatment. Our previous investigation demonstrated the important role of m^6^A modification in RA progression and the contribution of *TGM2* mRNA m^6^A modification in the aberrant function of RA‐FLS.^5^ In this exploration, we verified the m^6^A modification of *TGM2* mRNA in another primary RA‐FLS. In addition, WTAP was demonstrated to regulate *TGM2* mRNA m^6^A modification and expression. Interestingly, WTAP‐mediated TGM2/NF‐κB/Myc axis and Myc‐mediated WTAP transcription suggested the formation of the TGM2/Myc/WTAP‐positive feedback circuit in activating NF‐κB signaling. These data added new knowledge to the regulatory function of WTAP and Myc in facilitating NF‐κB signaling.^39^, ^40^ More interestingly, OS could downregulate WTAP‐mediated TGM2 mRNA m^6^A modification and Myc‐mediated WTAP transcription to restrain the activation of the TGM2/Myc/WTAP‐positive feedback loop. Importantly, OS regulated key effectors identified in the dysregulated network to delay RA progression and improved the anti‐RA effect of MTX in the CIA mouse model without obvious side effects, suggesting OS as an effective anti‐RA effect drug candidate. Moreover, bioinformatics analysis verified the positive association between TGM2 expression and FLS infiltration in RA patients and confirmed the tight connection between dysregulated genes involved in the OS‐regulated molecular network and clinical characteristics of RA patients, further supporting the conclusions drawn from RA‐FLS. In the present work, we speculated that OS might directly target RELA to affect the proposed working model in our work. However, we notice the limitation that more evidence should be provided to confirm RELA as a target of OS.

RA‐ILD is regarded as extra‐articular manifestations associated with increased mortality of RA patients^41^ and detected in up to 60% of patients with RA on high‐resolution computed tomography, serving as a leading cause of illness and death in patients with RA.^22^ Accumulating evidence showed that the RA disease activity was associated with an increased risk of developing RA‐ILD.^38^, ^42^, ^43^ RA‐ILD was inducible in several well‐established animal models, such as the CIA mouse model.^44^ In humans, usual interstitial pneumonia is the most common type of RA‐ILD and is the archetype of progressive fibrosis.^45^, ^46^ Interstitial lung lesions in the CIA mouse model resembled usual interstitial pneumonia in humans and were mostly located in the subpleural region of the lungs with the infiltration of inflammatory cells that were mainly CD11b^+^ macrophages of the M2 phenotype.^47^ TGM2, CD206, and CD163 were known as markers of M2 macrophages that positively correlated with fibrosis.^48^, ^49^, ^50^, ^51^ In our work, we showed the inhibitory effect of OS on the aggregation of CD11b^+^ macrophages in the subpleural region with interstitial lung lesions of CIA models. Moreover, the in vitro studies also verified that OS suppressed the proliferation and polarization of CD11b^+^ M2 macrophages by reducing TGM2 expression. Previous studies also demonstrated that TGM2 mediated the polarization of M2 macrophage and cell proliferation,^17^, ^52^ further supporting the findings obtained in our investigation. Interestingly, the reduced number of infiltrated M2 macrophages and inhibited M2 polarization might contribute to the suppression of RA‐ILD.^53^ Moreover, systemic inflammation was associated with an increased risk of developing RA‐ILD,^42^ and attenuating general inflammation and disease activity could improve lung function in RA‐ILD patients.^54^ Therefore, OS might alleviate RA‐ILD by mitigating the aggregation of CD11b^+^ macrophages in lungs of CIA models and downregulating systemic inflammation, further suggesting OS as a drug candidate,^55^, ^56^, ^57^ which might be beneficial to the treatment of RA‐ILD.

In summary, this study proved that OS showed an additive effect with MTX and suppressed proliferation, migration, and invasion of RA‐FLS by impairing the activity of TGM2‐mediated NF‐κB signaling (Figure 8G). In addition, our present investigation provided a new feedback circuit consisting of TGM2, Myc, and WTAP in the facilitation of RA progression and elucidated an original mechanism that Myc transactivated WTAP expression to mediate *TGM2* mRNA m^6^A modification. Moreover, OS impaired the TGM2/Myc/WTAP‐facilitated NF‐κB signaling and the proliferation as well as the polarization of CD11b^+^ M2 macrophages to control RA and RA‐ILD progression. This work delineates a theory for the clinical translation of OS as an effective anti‐RA and anti‐RA ILD drug without toxicity and emphasizes the potential application of TGM2 as a target for RA treatments.

## 4 MATERIALS AND METHODS

### 4.1 Cell cultures and chemical compounds

Human primary RA‐FLS with identification (Figure S8) and MH7A cell line adopted in this investigation were obtained and previously described in our publication.^5^ Mice macrophage RAW 246.7 and human monocyte THP‐1 cell line were purchased from Porcell corporation with STR verification. Ethics approval from the Ethics Committee of the hospital was obtained (No. 2020‐007). Passages 3 to 8 of the primary RA‐FLS and MH7A cells were cultured in Dulbecco's modified Eagle medium (DMEM) containing 10% fetal bovine serum (FBS) at a 37°C incubator with 5% CO_2_. Cells were routinely detected to be out of mycoplasma contamination. OS was purchased from PUSH Biotechnology Corporation (CAS: 484‐12‐8). MTX was purchased from Yuanye Biotechnology Corporation (CAS: 59‐05‐2). TNFα (40 ng/mL) purchased from PeproTech (300‐01A‐50) was applied for cell culture in the indicated experiments.

### 4.2 Cell transfections

Plasmids (ov‐TGM2, ov‐RELA) were established by GeneChem Corporation. TGM2 siRNAs (si‐TGM2), WTAP siRNAs (si‐WTAP), and Myc siRNAs (si‐Myc) were designed by RiboBio Corporation (Table S1). Following the manufacturer's protocol, Lipofectamine 3000 was applied for the transfection of plasmids and siRNAs into RA‐FLS.

### 4.3 Cell viability assay

Cell viability assays were implemented in RA‐FLS and RAW246.7. Cells were then added by CCK‐8 reagent (K1018; APExBIO Corporation; 10 μL) for 1‐h incubation and subjected to the detection of absorbance values by a microplate reader (BioTek) at 450 nm as we previously reported.^58^

### 4.4 Flow cytometry

RA‐FLS and RAW246.7 were used for flow cytometry analysis. For analyses of cell cycle, a cell cycle detection kit (CCS012; Multi Sciences) was used for experiments. Briefly, RA‐FLS were treated in a solution containing PI and permeabilization reagent for 30 min at 25°C. Then, the cells were applied for cell cycle analysis by flow cytometry. For cell surface marker staining, RAW246.7 cells were co‐stained with PECY7‐conjugated F480, FITC‐conjugated CD11b, APC‐conjugated CD206, BV421‐conjugated CD163, and Zombie Violet dye purchased from Biolegend corporation. The detailed information on antibodies is shown in Table S2. Then, the percentages of marker‐positive cells were measured by flow cytometry.

### 4.5 EdU assay

EdU assay was performed with a kit (K1077; APExBIO Corporation). Briefly, RA‐FLS were cultured with EdU solution (10 μM) for 4 h and were then subjected to fixation and permeabilization. After the addition of the reaction cocktail for another 30‐min incubation, cells were photographed under a fluorescence microscope. Image J software was applied for quantification.

### 4.6 Wound healing assay

RA‐FLS were cultured to obtain a confluent monolayer in a cell culture plate. Wounds were generated by scratching the cell monolayer with a pipette tip (200 μL). The scathing cells were then incubated in serum‐free DMEM after washing. At time points of 0, 24, and 48 h, wounds stained with crystal violet were recorded under a microscope for wound healing rate calculation.

### 4.7 Transwell assay

RA‐FLS suspension (100 μL) in serum‐free DMEM was added to the upper chamber of transwell, while a complete culturing medium (DMEM + 10% FBS) was added to the below chamber of transwell covered with or without matrigel. After 24–48 h incubation, the migrated (without matrigel) or invaded (with matrigel) cells were fixed and subjected to staining with crystal violet followed by image capture. Image J software was applied for quantification.

### 4.8 RNA‐sequencing analysis

RA‐FLS were cultivated with OS (20 μg/mL) for 48 h. Then, OS‐treated RA‐FLS and the controls were collected for total RNA extraction. RNA‐seq was carried out, and the data were analyzed as we described previously.^59^

### 4.9 PCR and qPCR

Total RNAs were isolated from RA‐FLS or macrophages for reverse transcription. Then, the generated cDNAs were subjected to PCR/qPCR with primers. The 2^−ΔΔCt^ method was applied for determining the relative mRNA expression for qPCR.

### 4.10 Western blot analysis

RA‐FLS and RAW246.7 were collected and lysed for cellular protein extraction and quantification. Total proteins were then subjected to separation, transfer, and incubation with specific antibodies. The detailed information on antibodies is shown in Table S2. Protein band detection was performed with a chemiluminescence method, and ChemiDocTM XRS+ (Bio‐Rad) was adopted for image capturing.

### 4.11 Immunofluorescence

RA‐FLS seeded on plates or tissue‐freezing medium‐embedded lung tissues were fixed, permeabilized, and treated with specific antibodies. The detailed information on antibodies is shown in Table S2. Then, RA‐FLS and lung tissues were subjected to co‐staining with DAPI, and the images were captured with a fluorescence microscope.

### 4.12 Methylated RNA immunoprecipitation

MeRIP was conducted with a MeRIP m^6^A kit (C11051‐1; RIBOBIO) described in our previous publication.^5^ Briefly, total RNAs extraction was performed followed by the fragmentation of the extracted RNAs into approximately 200 nucleotides. Then, the fragments were immunoprecipitated with m^6^A antibody‐conjugated magnetic beads. The eluted RNAs were purified and subjected to reverse transcription followed by qPCR and/or PCR. IgG was adopted as a negative control.

### 4.13 Chromatin immunoprecipitation

ChIP was performed with an Enzymatic Chromatin IP Kit (9003; CST). Following the manufacturers’ instructions, chromatin was crosslinked and extracted from RA‐FLS. Then, the extracted chromatin was applied for fragmentation followed by immunoprecipitated with specific antibody‐conjugated magnetic beads. The eluted DNA was subjected to purification and analyzed by qPCR and/or PCR. IgG was adopted as a negative control.

### 4.14 Cellular thermal shift assay

The assay was performed according to a previous study.^60^ Briefly, RA‐FLS were harvested for cell lysate preparation, and the soluble fraction was separated from the cell debris by centrifugation at 20,000 × g for 20 min at 4°C. The cell lysates were diluted with buffer harboring protease inhibitor cocktail and divided into two aliquots, with one aliquot being incubated with OS and the other aliquot being incubated with solvent (control) for 30 min at room temperature. Then, the lysates were divided into smaller (30 μL) aliquots and heated individually at 40, 45, 50, 55, 60, 65, 70, and 75°C for 3 min followed by cooling for 3 min at room temperature. The heated lysates were then subjected to centrifugation at 20,000 × g for 20 min at 4°C, and the supernatants were collected for western blot analysis.

### 4.15 In vivo animal study

These experiments and protocols were approved by the Animal Ethical Committee of Peking University Shenzhen Hospital (No. 2021–118). All experiments adhered to all relevant regulatory standards. CIA mouse model was established with bovine type II collagen in specific pathogen free (SPF) grade male DBA/1 mice aged 6–8 weeks (18–22 g) purchased from Shanghai SLAC Experimental Animal Corporation. All animals were maintained under an SPF environment and controlled conditions of a 12/12 light/dark cycle with an ambient temperature of 22–25°C for ensuring the good health of laboratory animals. Mice were randomly assigned into five groups with 10 mice in each group: the control group, the CIA model group, the MTX‐treated group, the OS‐treated group, and the MTX plus OS‐treated group. The primary immunization was performed on Day 0, and the enhanced immunization was performed on Day 21. The degree of arthritis in the limbs of the mice was scored as follows: normal (0), mild redness (1), moderate redness (2), severe swelling (3), and joint deformation and stiffness (4). The sum of the joint scores of the mouse limbs was defined as the arthritis score of each mouse. Based on the effective anti‐inflammatory concentration applied in previous studies,^12^, ^61^ OS (50 mg/kg) and the positive control drug MTX (23 mg/kg) were dissolved with solvent and intraperitoneally injected into the CIA models three times per week to observe the effects of OS and MTX on arthritis score and incidence of arthritis. After the mice were euthanized, the joints, lungs, livers, kidneys, and serum of the mice were harvested for H&E staining, immunofluorescence, immunohistochemistry, and enzyme‐linked immunosorbent assay (ELISA). Detailed histopathological scores of arthritis severity were blindly measured by experimenters as described in our previous study.^10^

### 4.16 ELISA

ELISA was conducted with purchased kits (2036T; Chondrex; WEA133Mu; Cloud‐Clone) according to the manufacturer's instructions. Standards or serum samples were added to the microplate pre‐coated with an antibody specific to type II collagen IgG or TNFα. After washing, a biotin‐conjugated antibody specific to type II collagen IgG or TNFα was added to the microplate. Then, TMB substrate solution is added to the microplate after washing and the enzyme‐substrate reaction was terminated by the addition of sulphuric acid solution. The detection of absorbance values was performed by a microplate reader (BioTek) at 450 nm.

### 4.17 Immunohistochemistry

Paraffin‐fixed joint tissues (4 μm) were deparaffinized and rehydrated followed by antigen retrieval with citrate buffer, the eradication of endogenous peroxidase activity by H_2_O_2_, and the blocking of non‐specific antigens by goat serum. Tissues were then treated with specific antibodies. The detailed information on antibodies is shown in Table S2. The staining signals were measured by DAB substrate and were blindly evaluated by pathologists.

### 4.18 Bioinformatics analysis

The RNA‐seq and clinicopathological data of synovium tissues and FLS were gained from PEAC RNA‐seq database (https://peac.hpc.qmul.ac.uk/) and GEO database (http://www.ncbi.nlm.nih.gov/geo/). GSEA, ssGSEA, correlation analysis, and differential expression analysis were conducted as previously described in our studies.^59^ Samples were assigned to high and low TGM2 groups based on the median expression value. For molecular docking, the structure of RELA protein (PDN: 3QXY) was downloaded from PDB database. Then, the sdf file of OS was downloaded from the PubChem database. CDOCKER docking module in DS software was applied for docking calculation and analysis.

### 4.19 Statistical analysis

RStudio and SPSS 21.0 were adopted for data analyses. The data from at least five independent experiments are expressed as the mean ± SD. The Wilcoxon rank‐sum test and the Student's two‐tailed *t*‐test were adopted for comparing the difference between the two groups. The one‐way ANOVA with LSD‐*t*‐tests was applied for comparing the difference among multiple groups. The Spearman's rank correlation test was used for conducting the correlation analyses. *p*‐values < 0.05 were significant.

## AUTHOR CONTRIBUTIONS

Q.W.: Conceptualization, validation, funding acquisition, and writing–review and editing. X.L. and J.C.: Conceived and designed this study, writing–original draft, investigation, data analyses, and funding acquisition. C.T., L.L., and J.H.: Investigation and data analyses. The final manuscript has been approved by all authors.

## CONFLICT OF INTEREST STATEMENT

The authors declare no competing interest.

## ETHICS STATEMENT

Human specimens: RA patients’ sample acquisitions were approved by the Ethics Committee of Peking University Shenzhen Hospital (No. 2020‐007). Written informed consent has been obtained from all patients.

Mice: All the procedures were carried out following the Guide for the Care and Use of Laboratory Animals and approved by the Animal Ethics Committee of Peking University Shenzhen Hospital (No. 2021–118).

## Supporting information

Supporting Information

## Data Availability

The data that support the findings of this study are available on request from the corresponding author.
