# Supplementary material for: Osthole regulates N6‐methyladenosine‐modified TGM2 to inhibit the progression of rheumatoid arthritis and associated interstitial lung disease
Source: MedComm (2020). 2023 Feb 22;4(2):e219. doi: 10.1002/mco2.219 (PMC9945862; doi:10.1002/mco2.219)
Supplement: Supplementary file 1 — Supporting Information [file MCO2-4-e219-s001.doc]

Supplementary material for

**Osthole regulates N6-methyladenosine-modified TGM2 to inhibit the progression of rheumatoid arthritis and associated interstitial lung disease**

Xian Lin ^1, 2, #, *^, Jian Chen ^1, 2, #^, Cheng Tao ^3^, Lianxiang Luo ^4, 5^, Juan He ^1, 2^, Qingwen Wang ^1, 2, *^

^1^ Department of Rheumatism and Immunology, Peking University Shenzhen Hospital, Shenzhen, China

^2^ Institute of Immunology and Inflammatory Diseases, Shenzhen Peking University-The Hong Kong University of Science and Technology Medical Center; Shenzhen Key Laboratory of Inflammatory and Immunology Diseases, Shenzhen, China

^3^ School of Pharmacy, Guangdong Medical University, Dongguan, China.

^4^ The Marine Biomedical Research Institute, Guangdong Medical University, Zhanjiang, China.

^5^ The Marine Biomedical Research Institute of Guangdong Zhanjiang, Zhanjiang, China.

^#^ These authors contributed equally to this work.

^*^ **Correspondence authors:**

Xian Lin and Qingwen Wang: Department of Rheumatism and Immunology, Peking University Shenzhen Hospital, Shenzhen Peking University-The Hong Kong University of Science and Technology Medical Center, 1120 Lianhua Road, Futian District, Shenzhen 518035, Guangdong, China. Email: linxiangabriel@fjmu.edu.cn (Xian Lin), [wqw_sw@163.com](mailto:wqw_sw@163.com) (Qingwen Wang). Phone: +86-0755-83923333.

**This file includes:** Figure S1-8, Table S1 and S2.


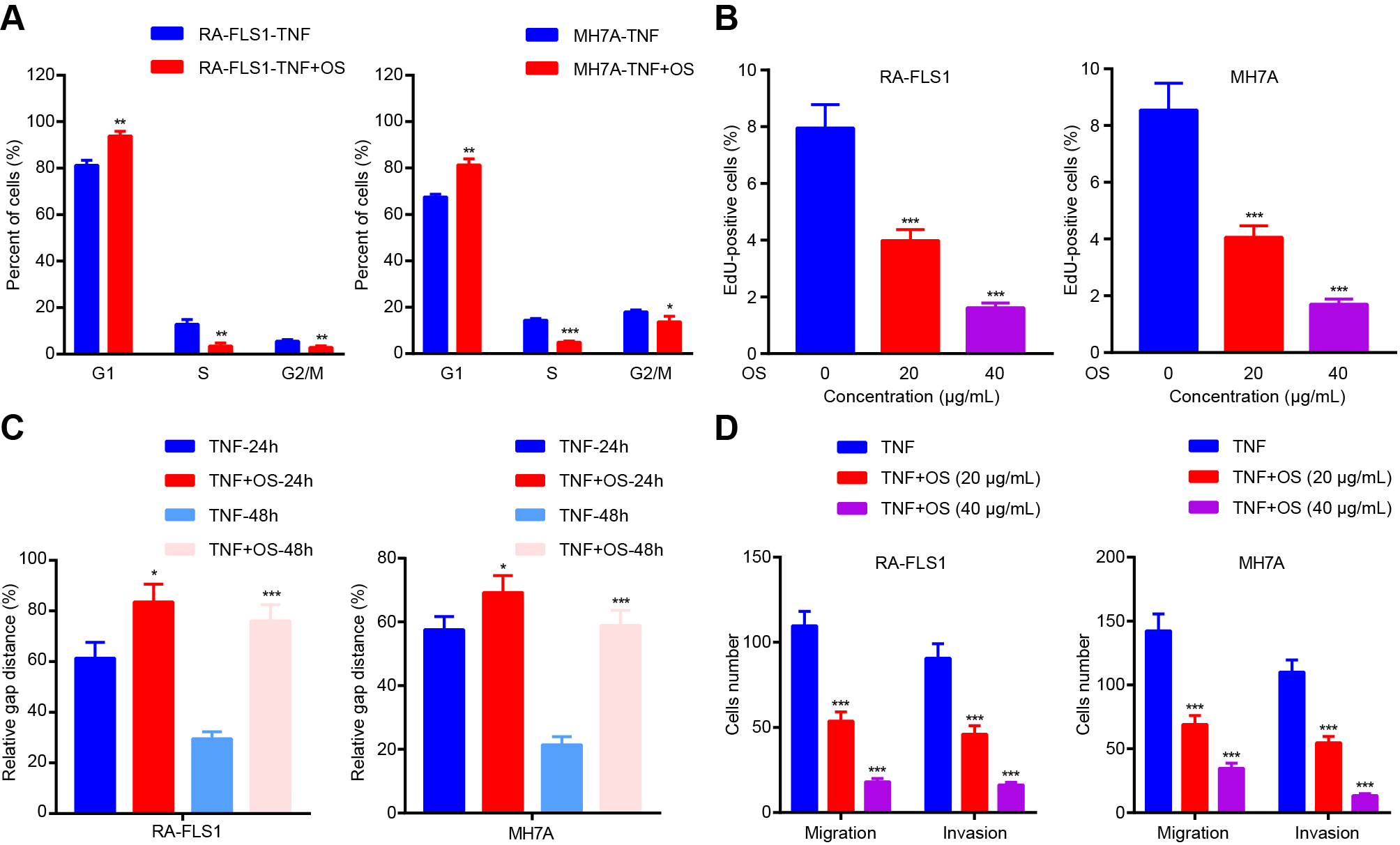


**Figure S1.** **Osthole inhibits proliferation, migration and invasion of RA-FLS.** (A) The cell cycle analyses were conducted by flow cytometry in osthole (0 and 20 μg/mL for 48 h)-treated RA-FLS and the controls. (B) EdU assays were applied for detecting DNA replication in osthole (0, 20, and 40 μg/mL for 48 h)-treated RA-FLS and the controls. (C) Wound healing assays were adopted to present the migration ability of osthole (0 and 20 μg/mL for 48 h)-treated RA-FLS and the controls. (D) Transwell assays were adopted to present the migration and invasion ability of osthole (0, 20, and 40 μg/mL for 48 h)-treated RA-FLS and the controls. ^*^ *P* < 0.05, ^**^ *P* < 0.01, and ^***^ *P* < 0.001 versus TNF-treated group.


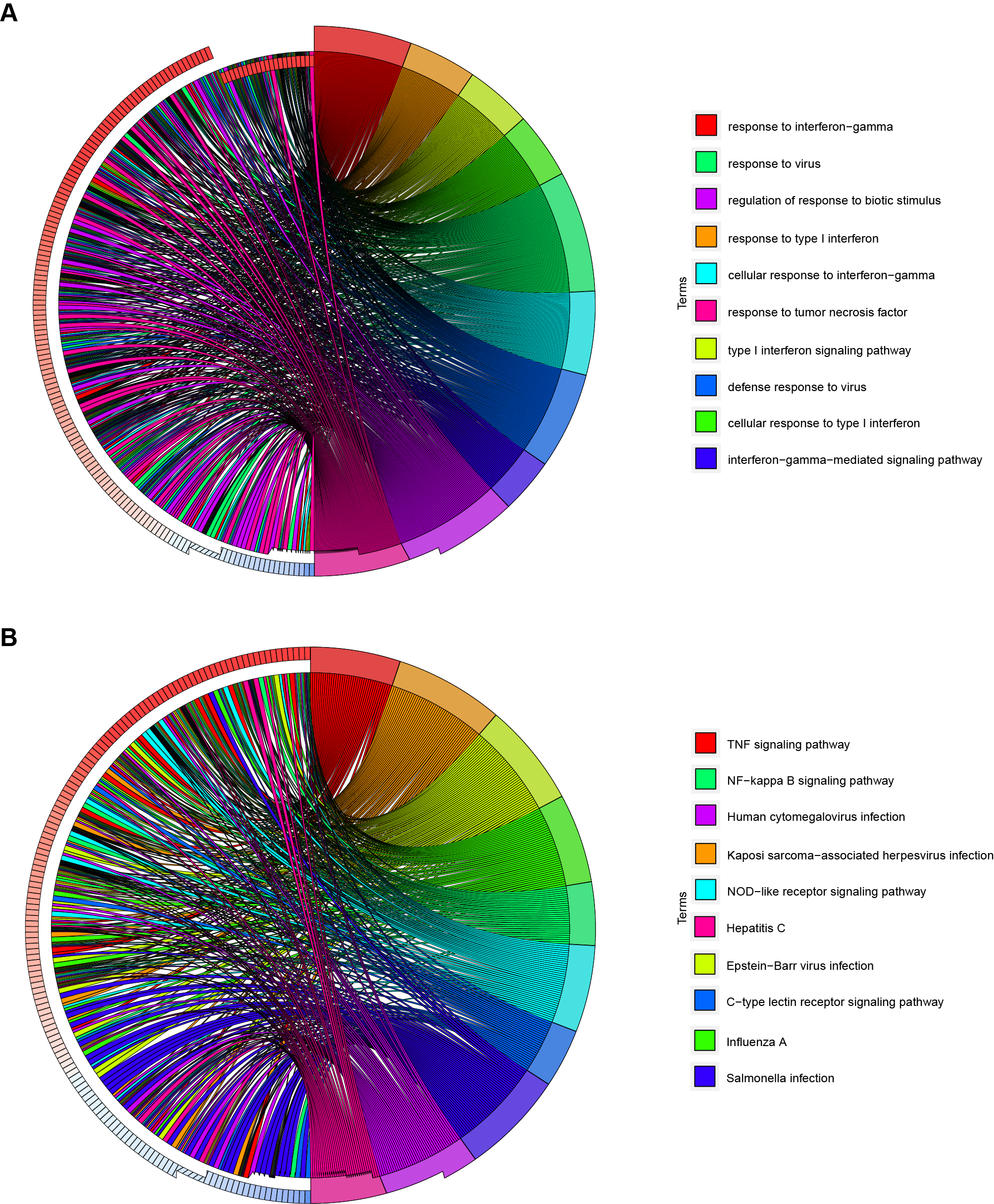


**Figure 2.** **Osthole potentially regulates TNF/NF-κB signaling based on RNA-seq analyses.** (A, B) Circos showing the top enriched GO (A) and KEGG (B) terms according to RNA-seq data of TNF-induced RA-FLS and the controls in GSE129486.


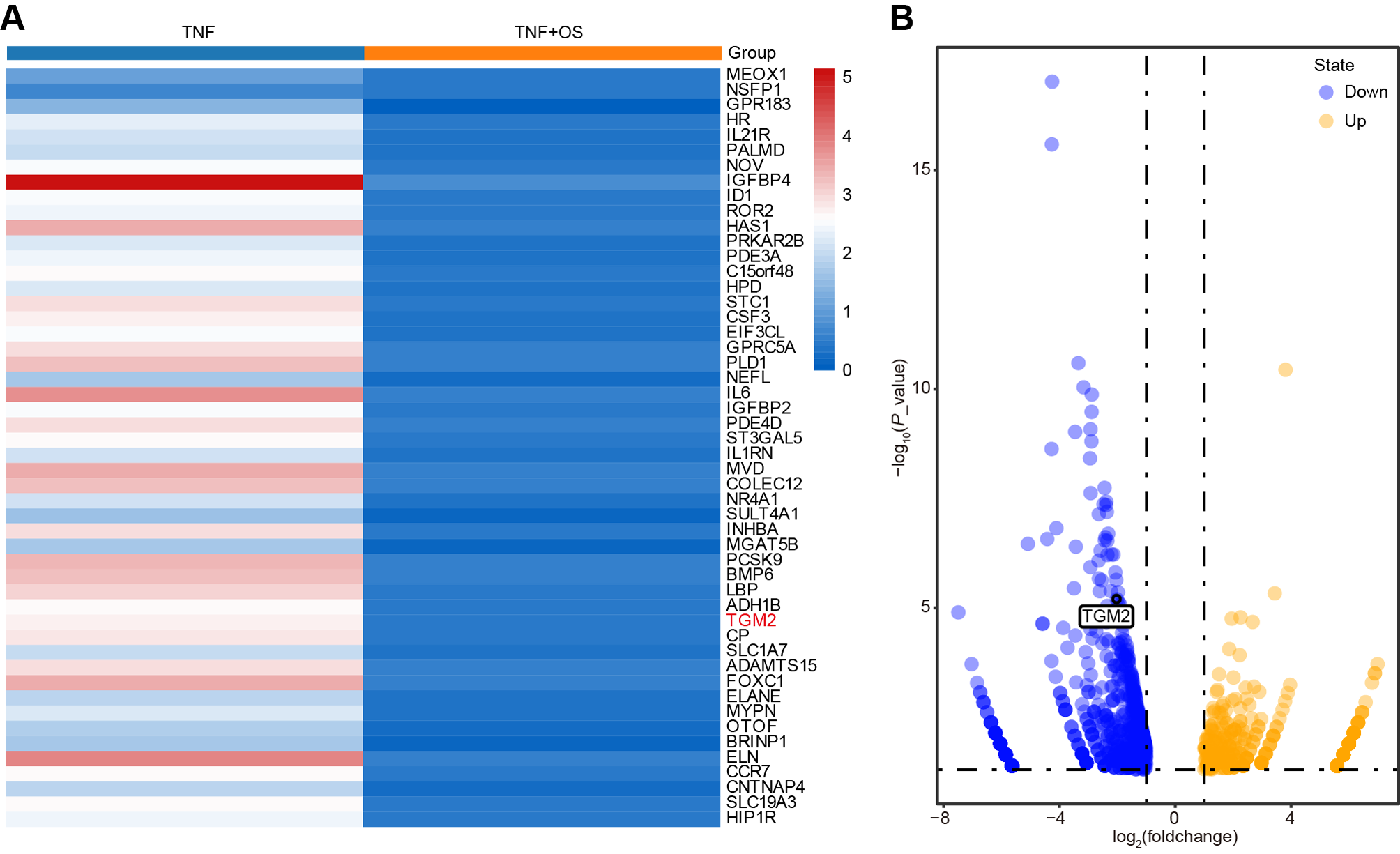


**Figure S3.** **Osthole suppresses TGM2 expression to attenuate the pathological phenotype of RA-FLS through modulating NF-κB signaling.** (A) A heatmap was plotted to show the top 50 differentially expressed genes between osthole-treated RA-FLS and the controls based on our RNA-seq data. (B) A volcano plot presenting differentially expressed genes between osthole-treated RA-FLS and the controls as per our RNA-seq data.


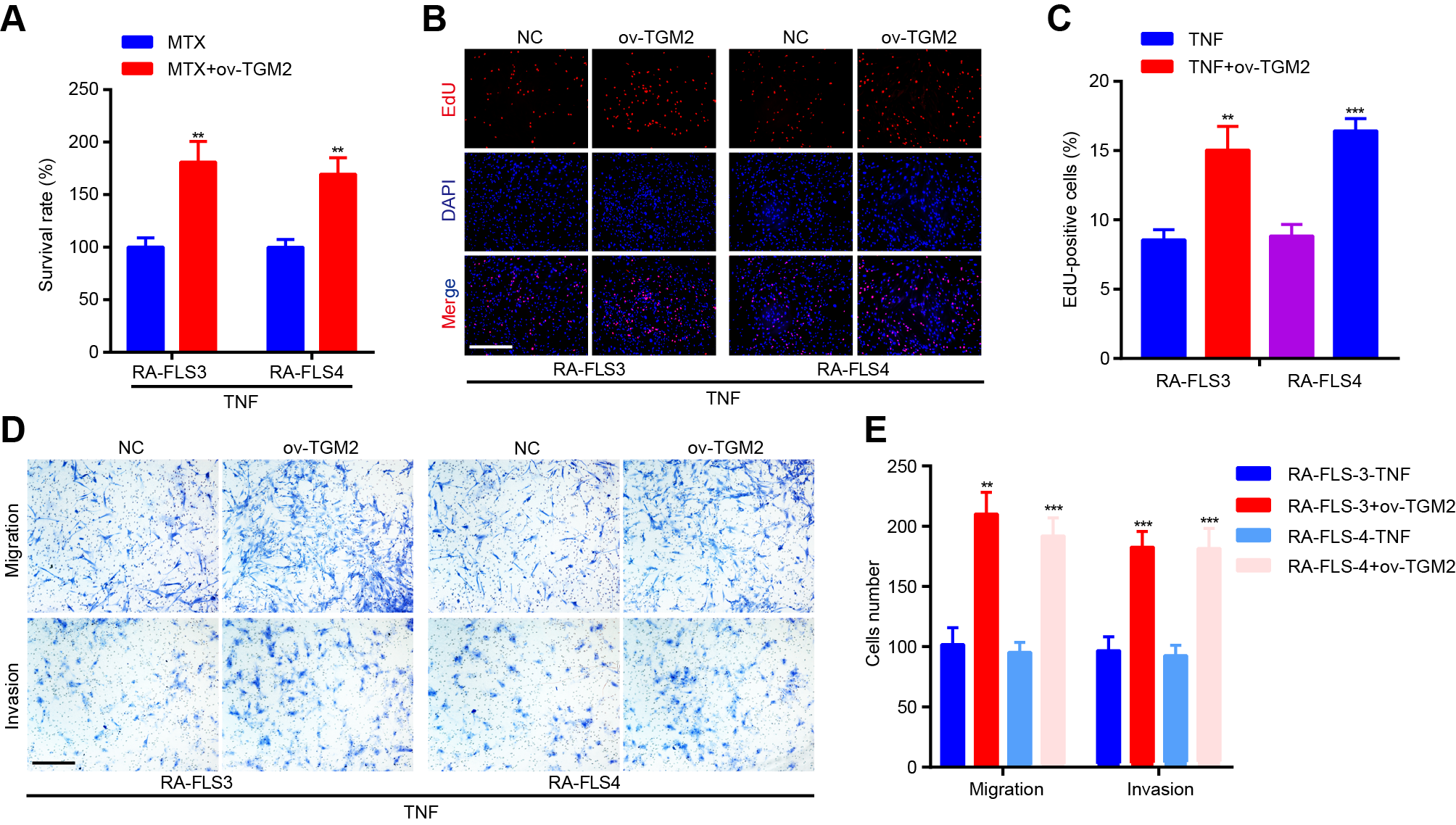


**Figure S4.** **TGM2 inhibits anti-RA effect of MTX and promotes the proliferation, migration, and invasion of RA-FLS.** (A) CCK8 assays were adopted to show the impact of TGM2 overexpression on anti-RA effect of MTX of RA-FLS. (B, C) EdU assays were applied to detect the effect of TGM2 overexpression on DNA replication of RA-FLS. Scale bar: 50 μm. (D, E) Transwell assays were adopted to determine the impact of TGM2 overexpression on RA-FLS migration and invasion ability. Scale bar: 50 μm. ^**^ *P* < 0.01, and ^***^ *P* < 0.001 versus TNF-treated group.


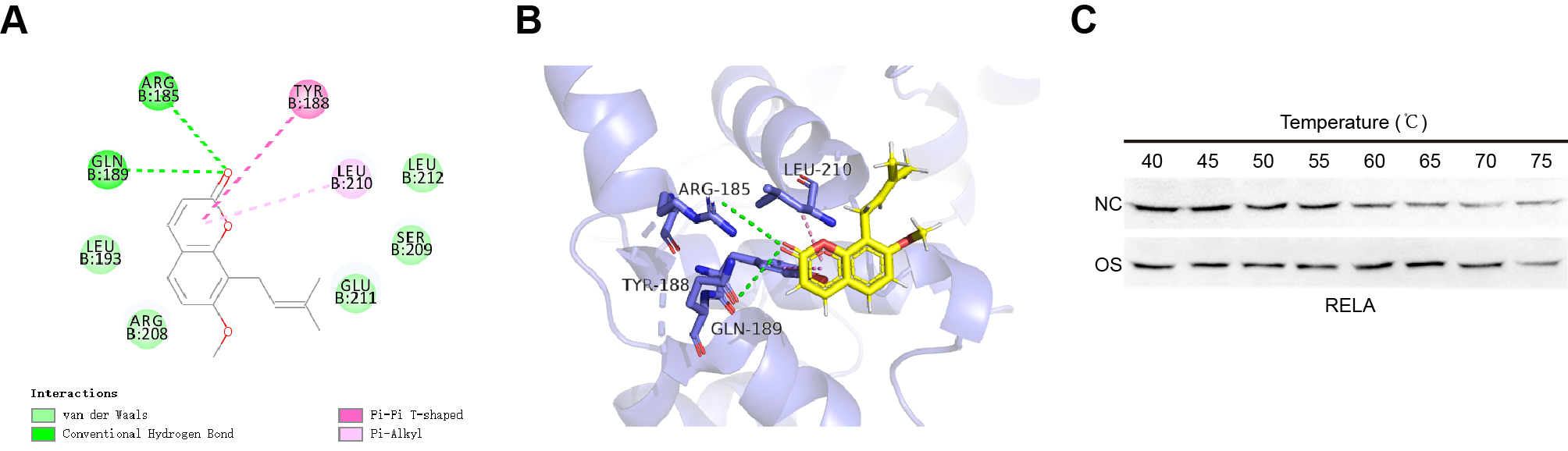


**Figure S5.** **Osthole might directly target RELA in RA-FLS.** (A) 2D diagram of CDOCKER docking results of osthole and RELA protein. (B) 3D interaction diagram of osthole with active residues of RELA protein. (C) A cellular thermal shift assay was performed to elucidate the effect of osthole on RELA protein.


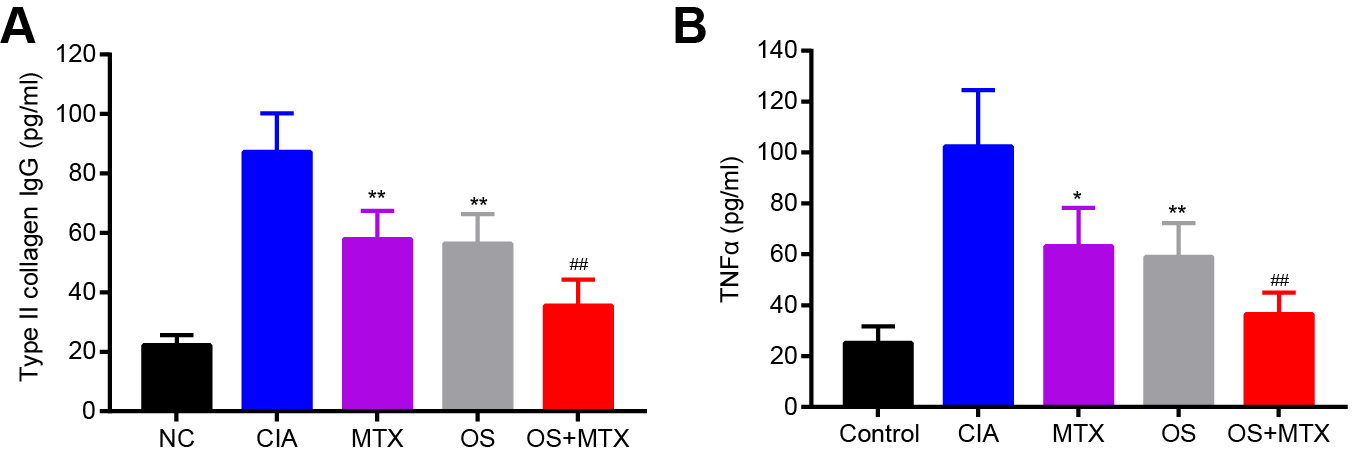


**Figure S6.** **The detection of anti-type II collagen IgG autoantibodies and inflammation-associated cytokine** **TNFα in mice models.** The CIA mouse model was constructed and randomly divided into control, CIA, MTX, osthole, and osthole combined with MTX treatment groups (*n* = 10 per group). Enzyme-linked immunosorbent assay was applied to measure the levels of anti-type II collagen IgG autoantibodies (A) and inflammation-associated cytokine TNFα (B) in mice models. ^*^ *P* < 0.05, and ^**^ *P* < 0.001 versus CIA group. ^##^ *P* < 0.01 versus MTX-treated group.


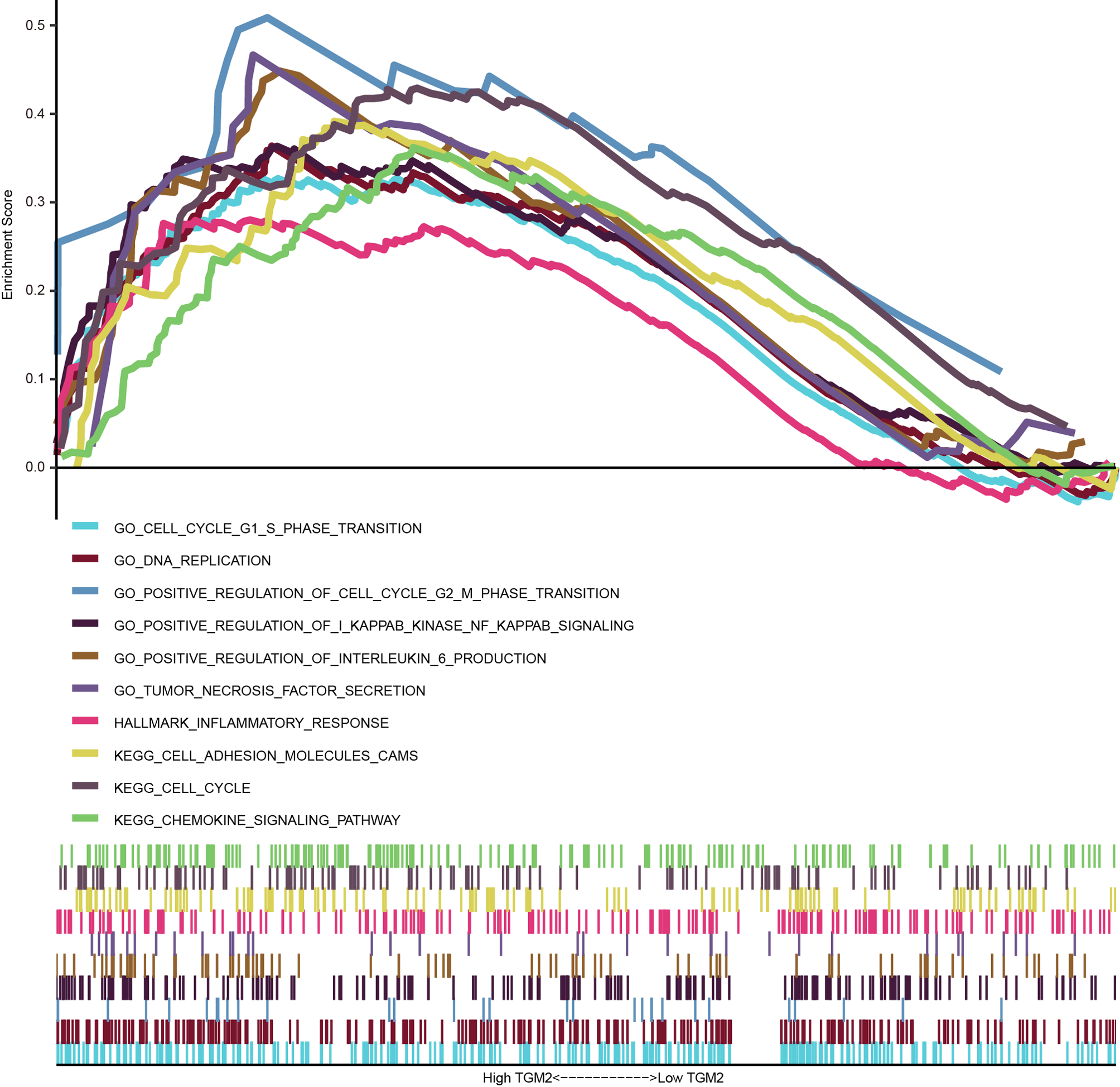


**Figure S7.** **Gene set enrichment analysis based on GSE109449 dataset.** Gene set enrichment analysis showing the differential enriched HALLMARK, GO, and KEGG terms between low TGM2 and high TGM2 groups as per GSE109449 dataset.


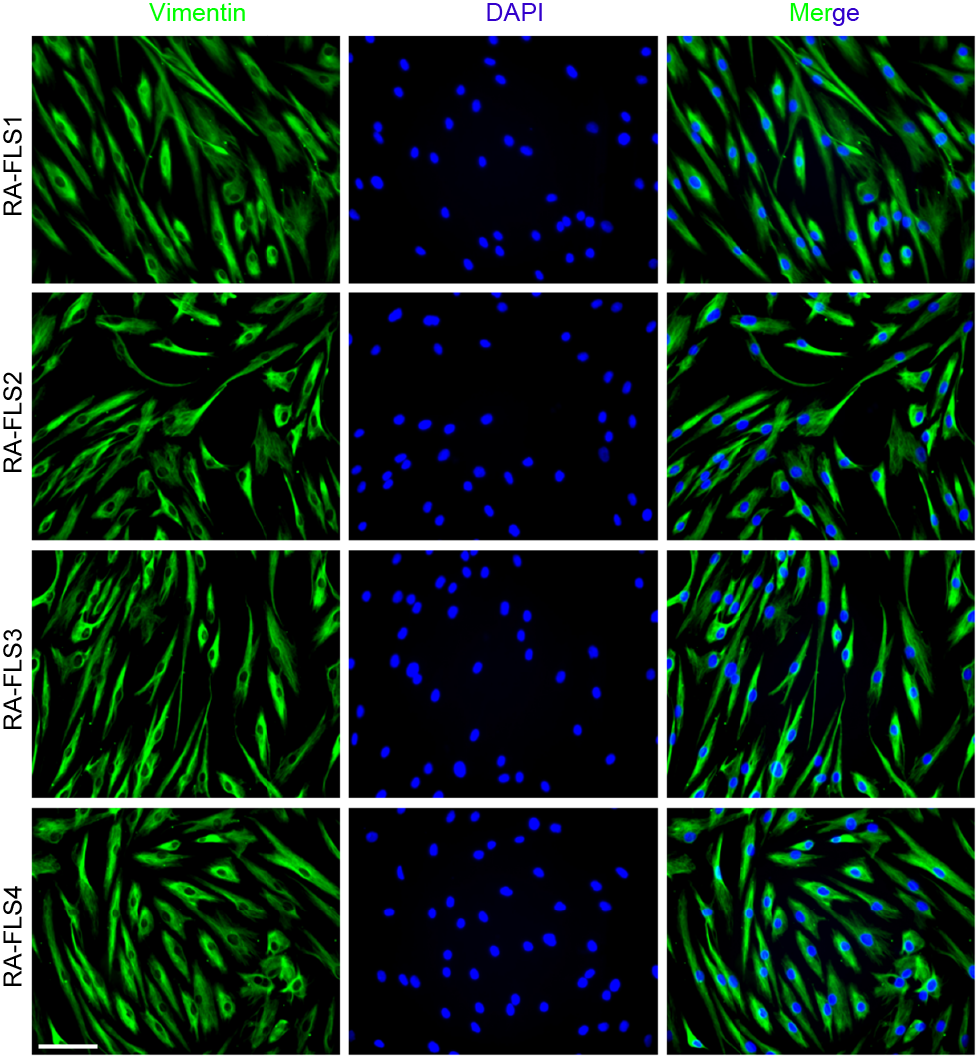


**Figure S8.** **The identification of primary RA-FLS by immunofluorescence staining of Vimentin.** Scale bar: 25 μm.

**Table S1. The sequences used in this study**

| Name | | Target sequences |
| --- | --- | --- |
| TGM2 siRNAs | 1 | 5’ GCAACCTTCTCATCGAGTA 3’ |
|  | 2 | 5’ GCCTGATCCTTCTAGATGT 3’ |
|  | 3 | 5’ GCATGAACATGGGCAGTGA 3’ |
| WTAP siRNAs | 1 | 5’ GGAACAGACTAAAGACAAA 3’ |
|  | 2 | 5’ CTAAGAGAGTCTGAAGAAA 3’ |
|  | 3 | 5’ GAAGCATATGTACAAGCTT 3’ |
| Myc siRNAs | 1 | 5’ GAGGAGACATGGTGAACCA 3’ |
|  | 2 | 5’ GGGTCAAGTTGGACAGTGT 3’ |
|  | 3 | 5’ CGACGAGACCTTCATCAAA 3’ |

**Table S2.** A list of antibodies used for FC, WB, IF, ChIP, and IHC

| Antibody | Cat. No | Company | Species | Application |
| --- | --- | --- | --- | --- |
| PE/CY7 anti-mouse F4/80 | 123114 | Biolegend | Rat | FC |
| FITC anti-mouse/human CD11b | 101206 | Biolegend | Rat | FC, IF |
| APC anti-mouse CD206 | 141708 | Biolegend | Rat | FC |
| BV421 anti-mouse CD163 | 155309 | Biolegend | Rat | FC |
| Myc | 18583S | CST | Rabbit | WB, ChIP |
| Myc | bs-4963R | bioss | Rabbit | IHC, IF |
| TGM2 | 15100-1-AP | Proteintech | Rabbit | WB, IHC, IF |
| CCNA2 | [18202-1-A](https://www.ptglab.com/products/ACTB-Antibody-60008-1-Ig.htm)P | Proteintech | Rabbit | WB, IHC |
| CCNB1 | [28603-1-A](https://www.ptglab.com/products/ACTB-Antibody-60008-1-Ig.htm)P | Proteintech | Rabbit | WB |
| Vimentin | 10366-1-AP | Proteintech | Rabbit | WB, IHC |
| N-ca | 22018-1-AP | Proteintech | Rabbit | WB |
| E-ca | 20874-1-AP | Proteintech | Rabbit | WB |
| WTAP | 10200-1-AP | Proteintech | Rabbit | WB, IHC, IF |
| CD163 | 16646-1-AP | Proteintech | Rabbit | WB |
| β-actin | 66009-1-Ig | Proteintech | Mouse | WB |
| p-NF-κB | AF2006 | Affinity | Rabbit | WB |
| NF-κB/RELA | AF5006 | Affinity | Rabbit | WB, IF |
| ABCG2 | AF5177 | Affinity | Rabbit | WB, IHC |
| GAPDH | [AF7021](http://www.affbiotech.com/goods-6289-AF7021-GAPDH_Antibody.html) | Affinity | Rabbit | WB |

FC: flow cytometry, WB: western blot, IF: immunofluorescence, ChIP: chromatin immunoprecipitation, IHC: immunohistochemistry.
